# Deuterium Magnetic Resonance Imaging and the Discrimination of Fetoplacental Metabolism in Normal and L-NAME-Induced Preeclamptic Mice

**DOI:** 10.3390/metabo11060376

**Published:** 2021-06-10

**Authors:** Stefan Markovic, Tangi Roussel, Michal Neeman, Lucio Frydman

**Affiliations:** 1 Department of Chemical and Biological Physics, Weizmann Institute of Science, Rehovot 7610001, Israel; stefan.markovic@weizmann.ac.il; 2 Center for Magnetic Resonance in Biology and Medicine, 13385 Marseille, France; tangi.roussel@gmx.com; 3 Department of Biological Regulation, Weizmann Institute of Science, Rehovot 7610001, Israel; michal.neeman@weizmann.ac.il

**Keywords:** pregnancy, fetal metabolism, placental transport, preeclampsia, deuterium metabolic imaging

## Abstract

Recent magnetic resonance studies in healthy and cancerous organs have concluded that deuterated metabolites possess highly desirable properties for mapping non-invasively and, as they happen, characterizing glycolysis and other biochemical processes in animals and humans. A promising avenue of this deuterium metabolic imaging (DMI) approach involves looking at the fate of externally administered ^2^H_6,6′_-glucose, as it is taken up and metabolized into different products as a function of time. This study employs deuterium magnetic resonance to follow the metabolism of wildtype and preeclamptic pregnant mice models, focusing on maternal and fetoplacental organs over ≈2 h post-injection. ^2^H_6,6′_-glucose uptake was observed in the placenta and in specific downstream organs such as the fetal heart and liver. Main metabolic products included ^2^H_3,3′_-lactate and ^2^H-water, which were produced in individual fetoplacental organs with distinct time traces. Glucose uptake in the organs of most preeclamptic animals appeared more elevated than in the control mice (*p* = 0.02); also higher was the production of ^2^H-water arising from this glucose. However, the most notable differences arose in the ^2^H_3,3′_-lactate concentration, which was ca. two-fold more abundant in the placenta (*p* = 0.005) and in the fetal (*p* = 0.01) organs of preeclamptic-like animals, than in control mice. This is consistent with literature reports about hypoxic conditions arising in preeclamptic and growth-restricted pregnancies, which could lead to an enhancement in anaerobic glycolysis. Overall, the present measurements suggest that DMI, a minimally invasive approach, may offer new ways of studying and characterizing health and disease in mammalian pregnancies, including humans.

## 1. Introduction

Fetal impairments are often associated with abnormal phenotypes such as intrauterine growth restriction and preeclampsia; these conditions are also known to substantially enhance the susceptibility of mother and child to subsequent chronic illnesses [1,2]. Whilst ultrasound presents the gold standard in pregnancy monitoring, these measurements struggle to achieve high contrast and metabolic insight—limitations which often restrict the revelation of abnormal placental conditions to morphologically advanced pregnancy stages [3]. Magnetic resonance imaging (MRI) methods are much more versatile and richer in the information they can provide [4,5,6]. A diversity of water-based contrasts including T1, T2 and diffusion weightings endow MRI with an ability to highlight abnormalities in morphology and perfusion at an early stage [7,8,9,10,11,12,13,14,15,16,17,18,19]. This contrast can be further enriched if combined with the chemical specificity that derives from MR spectroscopy and/or spectroscopic imaging (MRS, MRSI) measurements [20,21]. Still, pregnancy examinations often struggle to assess how morphological changes observable by ultrasound or MRI, actually affect the physiological functions—foremost among these fetoplacental transport and metabolism. Mapping these in detail and with good quantitation is a non-trivial task that gets compounded by the safety precautions needed to prevent any potential damage to mother and fetus in an eventual exam [22]. This limits the use of classical metabolic and imaging techniques such as positron emission tomography (PET) or computed tomography (CT) [23,24]; following fetoplacental permeability and perfusion by established MRI methodologies such as contrast-enhanced imaging, is also compromised by safety concerns associated with these techniques’ injection of paramagnetic agents [25,26].

Thanks to the unprecedented sensitivity gains arising from nuclear hyperpolarization, ^13^C-based MRSI methodologies could offer a potential solution to this challenge [27,28,29,30]. The sensitivity of this approach has been recently shown to suffice for spatially mapping placental metabolism in pregnant rodents and in ex vivo human placentas [31,32,33,34]. An intrinsic limitation of hyperpolarized MRSI, however, rests in the rapid decay of its signals; this restricts the insight that can be gathered to a first passage perfusion, leading to “snapshots” that may fail to provide the full picture of metabolic activity. Alternative efforts to amplify metabolic MRI signatures have been done on the basis of saturation transfer approaches [35,36,37], including the use of chemical exchange saturation transfer of glucose (glucoCEST) to follow the uptake of glucose in rodents’ placentas [38]. Still, the spectral proximity between the glucose signal being saturated and the water resonance acting as reporter of this saturation implies that motions and inhomogeneities in regions such as the maternal pregnant abdomen, will limit the scope of such studies. These factors highlight the necessity for a reliable metabolic imaging tool, that is free from kinetic restrictions, and that can report on activity during pregnancy without safety hazards. 

Very recently, MRS and MRSI experiments targeting the hydrogen isotope deuterium, ^2^H, have been introduced as potential reporters of metabolism in heart and brain, in brain, pancreatic, liver, and lymphatic tumors, and in brown adipose tissue [39,40,41,42,43,44,45,46,47,48]. Such Deuterium Metabolic Imaging (DMI) approach involves the administration of deuterated precursors, such as glucose, which are as safe as their conventional protonated counterpart, and mapping the resulting deuterated products via ^2^H MRSI as a function of time. In particular, it was observed that, if selectively enriched ^2^H_6,6′_-glucose is administered either orally or by injection, it will distribute throughout the organism and convert into ^2^H_3,3′_-lactate as part of the glycolytic pathway, or as ^2^H-water (HDO) as a metabolic end-product of glycolysis, the Krebs cycle, and other pathways [39,40,41,49]. Brain studies also revealed the formation of ^2^H_4,4′_-glutamate and ^2^H_4,4′_-glutamine, as part of byproducts from the Krebs cycle in this organ [39,40]. 

Deuterium is similar in many respects to the protons normally targeted by MRS: it will barely affect metabolism, is non-radioactive, and can resolve various chemicals according to the distinct peaks that it originates along a spectral dimension. ^2^H has a negligible (0.01157%) natural concentration, meaning that its sole MRI background signal will come from the ≈10 mM HDO in tissues; this serves as “built-in ruler”, enabling the quantification of the metabolites’ concentrations, and their evolution as a function of time. An unfavorable ^2^H feature comes from its low gyromagnetic ratio, leading to an intrinsic sensitivity that is ≈100-fold lower than that of protons. This is partially compensated by ^2^H’s quadrupolar nature, which makes its T1 relaxation time very short, and enables an extensive signal averaging within relatively short periods of time. In combination with the sensitivity enhancement and spectral resolution provided by emerging high-field MRI scanners, this could transform DMI into a powerful tool for exploring metabolism under a diverse range of conditions [50].

The present study explores this potential, as applied to the investigation of glucose’s metabolism in control and preeclamptic pregnant mouse models. Preeclampsia is a condition often characterized by high blood-pressure and proteinuria. As a consequence, organs may often undergo damage and changes leading to an increase in anaerobic metabolism [51]. These effects are here assessed with the aid of DMI, on both control and Nω-Nitro-L-arginine methyl ester hydrochloride (l-NAME)-treated mice. l-NAME is a vasoconstrictor leading to symptoms that mimic preeclampsia [52,53,54]—including maternal hypertension, renal damage, proteinuria, and decreased fetal size. The possibility to detect the fate of selectively deuterated glucose in these animals’ abdomen, with acceptable spatial resolution and over extended (~150 min) timescales by ^2^H MRSI, was confirmed. Glycolytic activity could then be detected in placentas as well as in downstream spatially resolved fetal organs, including the fetal heart, liver and brain—all of these targets whose metabolism was inaccessible when assayed on rats by hyperpolarized ^13^C MRSI [31]. The dynamics of both the deuterated glucose transport and of its ensuing metabolic products were assessed in all the pregnant mice; when averaged over organs and over the measurements’ time series, it was observed that glucose uptake was higher in the l-NAME-treated preeclamptic animal models than in the controls—both in the placentas and the fetal organs. Even more notable differences could be observed in the rates of lactate accumulation, which reached higher levels in the preeclamptic models. These results were analyzed semi-quantitatively using kinetic models, leading to glycolytic rates which agreed well with literature reports based on radioactive tracers. The higher lactate levels observed in diseased over normal pregnancies is also in accordance with literature reports that suggested an enhanced anaerobic glycolysis in the former. DMI’s kinetic analyses, however, trace the lactate differential buildup to differences in the perfusion of the various metabolites out of the fetoplacental units. Overall, this study demonstrates that ^2^H magnetic resonance offers a minimally invasive metabolic tool for studying pregnancies, that can potentially detect preeclampsia.

## 2. Results

### 2.1. ^2^H magnetic Resonance on Pregnant Animals: Overall Features

**Figure 1:**
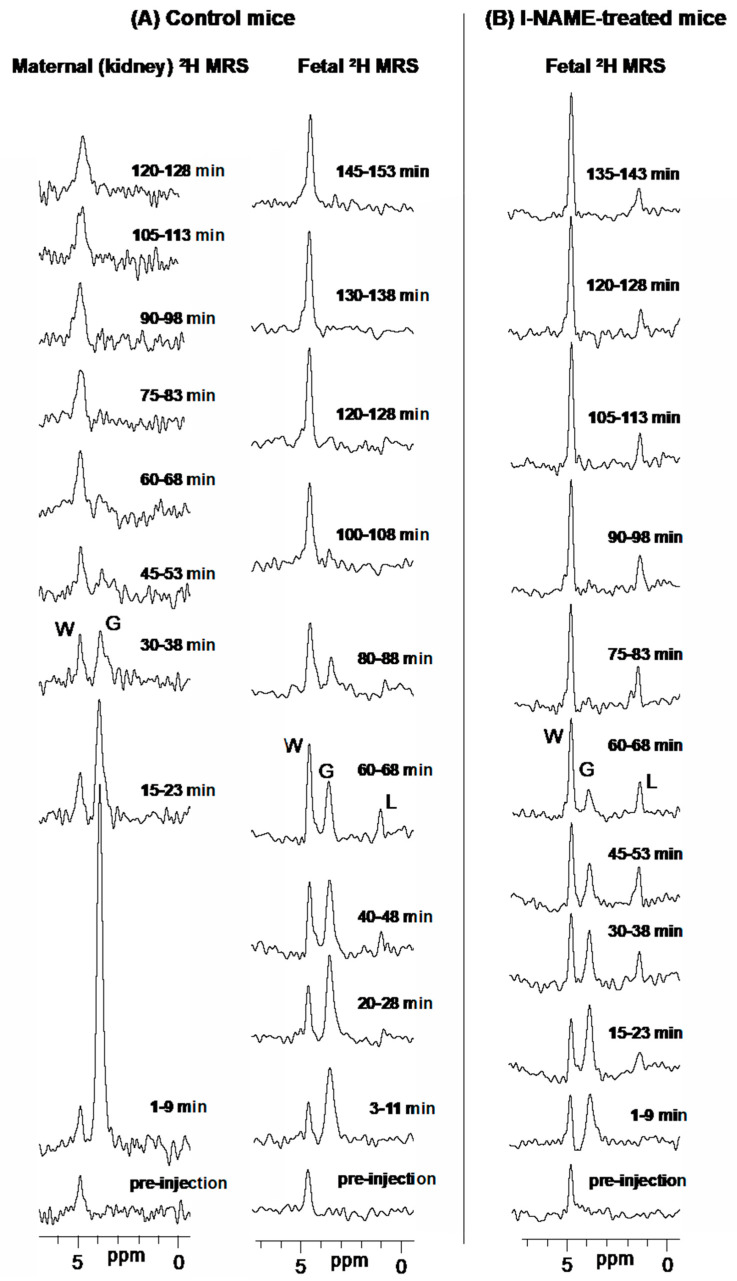
Representative ^2^H MRS time-series arising after intravenous administration of ^2^H_6,6′_-glucose to control (**A**) and l-NAME-treated (**B**) pregnant mice. The columns present localized spectra extracted from MRSI data from the indicated regions and at the indicated post-injection time-points (ranges indicate the extent of the MRSI signal averaging). Signals for ^2^H_6,6′_-glucose and its metabolic products water and lactate are indicated by letters G, W and L, respectively.

Bolus injections of ^2^H_6,6′_-glucose were followed with a time series of non-localized ^2^H MRS and slice selective 2D ^2^H MRSI experiments, recorded in an interleaved fashion. Figure 1A,B exemplify representative 1D localized ^2^H MRS data arising from the latter series, for l-NAME-treated and control pregnant animals. Prior to injection the ^2^H spectra show solely the naturally abundant ^2^H-water (“W”) signal at 4.8 ppm in all organs; following the glucose injection, the growth of a strong ^2^H_6,6′_-glucose peak at 3.9 ppm (“G”) in the maternal kidney, is clearly visible. Fetal (and fetoplacental) units evidence a slower emergence of a similar “G” peak; with time, the growth of a ^2^H-water and of an upfield peak at 1.3 ppm, can all be detected from the fetal regions with ample sensitivity and good spectral resolution. No other ^2^H resonances arising from compounds involved in either the glycolytic or other pathways, are detected. Following previous studies [39,40] we assign the 1.3 ppm to the methyl peak of ^2^H_3,3′_-lactate (“L”), with the di-deuteration of this compound’s methyl assumed to reflect a full transfer of the two deuterons that were initially bound glucose’s C_6_. The accumulation of an enhanced ^2^H-water peak as main final product is detected in the maternal kidney, but at no time is the 1.3 ppm peak detected anywhere but in the fetus or the placenta. The MRSI data also revealed a different temporal behavior for the control and for l-NAME-treated mice, with the latter’s lactate signals being generally stronger and longer-lived than in the controls. Occasionally, some non-localized ^2^H MRS sets (but not the lower sensitivity MRSI data) showed a background resonance at ≈1 ppm, which might be associated with abdominal fat.

### 2.2. DMI Reveals Differences between Normal and Preeclamptic Pregnancies 

**Figure 2:**
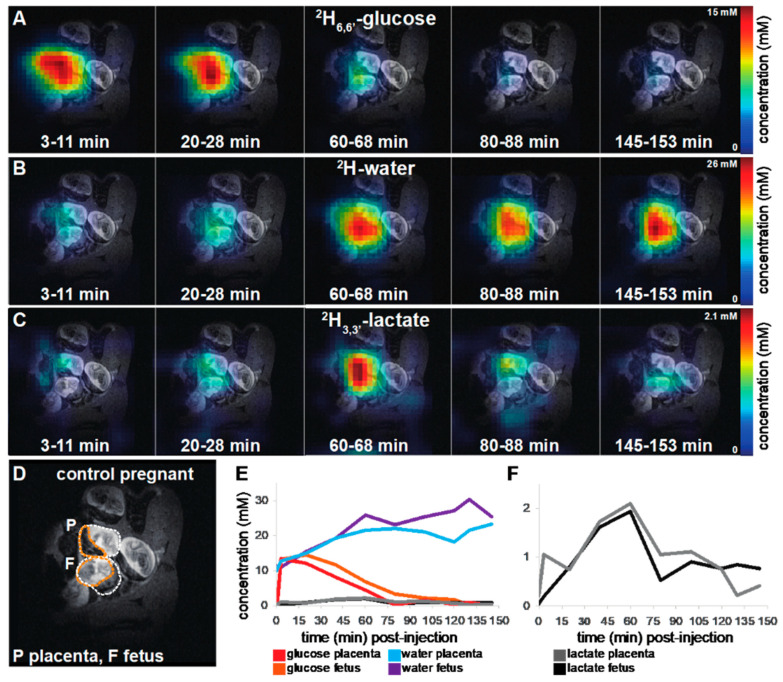
DMI data collected at the indicated stages following the intravenous administration of ^2^H_6,6′_-glucose to a control pregnant mouse. Ten ^2^H MRSI data sets were recorded over the time course of 161 min. Five of these data sets depicting the metabolic maps of ^2^H_6,6′_-glucose (**A**) and its metabolic products ^2^H-water (**B**) and ^2^H_3,3′_-lactate (**C**) are here shown for illustration purposes; the full time series can be found in Supplementary Figure S3. The anatomical ^1^H image on top of which all ^2^H data are shown is depicted in (**D**), highlighting in orange a placenta and a fetus, and in white dashes full fetoplacental units. Time traces for all metabolites (**E**) and a zoom into the low concentration ^2^H_3,3′_-lactate traces (**F**) are shown for the entire time series. Signal-to-noise values for the maximal signals in this image series were 16 for ^2^H_6,6′_-glucose, 20 for ^2^H-water and 3.4 for ^2^H_3,3′_-lactate. Non-interpolated images for this set are presented in Supplementary Figure S1.

Figure 2 shows the post-injection, time-dependent ^2^H MR images resolved at the ^2^H_6,6′_-glucose, ^2^H-water and ^2^H_3,3′_-lactate chemical shifts, overlaid over corresponding anatomical ^1^H images for a control mouse. Right after injection, ^2^H_6,6′_-glucose is detected for this scan mainly in the placenta and in the fetal liver (Figure 2A,E); as this peak gradually washes out from these organs, ^2^H-water is detected throughout the traces: prior to injection at its natural abundance level, and thereafter constantly increasing as a result of ^2^H_6,6′_-glucose’s metabolism—once again, mainly in the placenta and in fetal organs including brain and liver (Figure 2B,E). Lactate production can also be observed in these organs, reaching a maximum ~60 min after injection and subsequently washing out (Figure 2C,F). In total *n* = 9 control pregnant animals were scanned in this fashion following a bolus injection of ^2^H_6,6′_-glucose (see Materials and Methods for further details); the ^2^H MRSI images and metabolic kinetics observed in all these cases are summarized in Supplementary Figures S3–S11. Notice that: (i) In each of these scans the exact nature of the maternal organs and the number/orientation of fetoplacental units captured changed, as a result of the different positionings adopted by the organs within the field-of-views accessible by the surface coils employed (see Materials and Methods for further details). There is therefore a certain variability in the organs highlighted by each of the scans. (ii) In all cases the well-defined natural abundance ^2^H-water peak coupled to the nature of the experiments allowed us to translate MRSI peak areas into approximate analyte concentration levels; the ensuing plots are thus summarized as mM vs. min plots for individual metabolites and different organs in each animal. (iii) As lactate concentrations in the control animals were significantly lower than those of the other metabolites, the latter’s MRSI images were sometimes co-added over multiple experiments so as to improve their sensitivity; in such cases, fewer and further apart images are presented. In general, inspection of these data shows that following its initial injection, ^2^H_6,6′_-glucose perfuses into the maternal kidneys and the placentas within the time that it takes to collect the first ^2^H MRSI data set; in certain cases, accumulation into the animal’s bladder is also observed. Maximum concentrations in these organs varied between 10 and 40 mM, with a median of ~20 mM. This scattering is not unlike what was observed in hyperpolarized ^13^C MRSI in pregnant rats [31], where the efficiency of the agent’s perfusion showed a variability (presumably associated with the animals’ positioning). In the majority of scanned cases the ^2^H_6,6′_-glucose eventually perfused from the placentas into the fetal organs, reaching concentrations that were lower than in the initially perfused maternal kidneys or placentas—and maximizing 15–25 min post-injection. Lactate and ^2^H-water formation are also observed for a majority of placentas. ^2^H_3,3′_-lactate’s concentration peaks ca. 45 ± 20 min after injection and reaches concentrations of ~0.5–2 mM for these control animals (assuming that the lactate’s methyl peak is di-deuterated), washing out to baseline levels towards the end of the experiments. The ^2^H-water signal by contrast increased steadily throughout these measurements, reaching concentrations in the 15–30 mM range (Supplementary Figures S3–S11).

To explore an alternative to these relatively high dosage bolus injections, ^2^H MRSI datasets were acquired with an infusion-like protocol (*n* = 3). This involved a series of ~5–10 “microinjections” containing 25–50 μL of 2.6 M ^2^H_6,6′_-glucose each, applied on the control animals throughout ≥60 min. Data were constantly recorded over this time, up to a total of 120 min since its commencing. Supplementary Figures S12–S14 summarize these experiments. By contrast to the bolus injections these infusions did not lead to an initial “spike” in ^2^H_6,6′_-glucose, whose concentrations rarely exceeded ~5 mM throughout the entire kinetic time series. Interestingly, although ‘smeared’ over time, the glucose distribution throughout maternal and fetal organs was not markedly different from that observed in the bolus injection experiments. Further, the ^2^H_3,3′_-lactate levels reached in these infusions also did not differ significantly from those in the bolus injection experiments. ^2^H-water concentrations for these experiments were actually slightly higher than in the bolus administration, growing steadily until reaching 25–35 mM levels.

**Figure 3:**
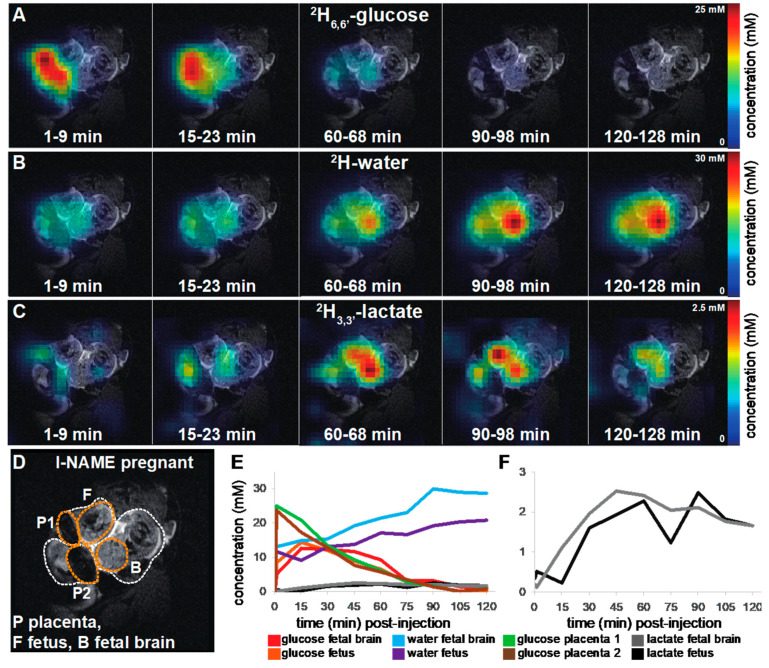
DMI data collected after intravenous administration of ^2^H_6,6′_-glucose to a pregnant mouse that has been pre-treated with l-NAME. Ten ^2^H MRSI images were recorded over the time course of 136 min. Five time-datapoints for metabolic maps of ^2^H_6,6′_-glucose (**A**) and its metabolic products ^2^H-water (**B**) and ^2^H_3,3′_-lactate (**C**) were selected for illustration purposes (the entire time series can be found in Supplementary Figure S16). The ^1^H anatomical reference image used as background is shown in (**D**), highlighting in dashed white circles three fetoplacental units and in orange two placentas, a fetal brain and a fetus. Time traces for all metabolites **(E**) and a zoom into the low concentration ^2^H_3,3′_-lactate traces (**F**) are shown for the entire time series. Signal-to-noise values for maximum signals in the time series are ~20 for ^2^H_6,6′_-glucose, 19 for ^2^H-water and 4.7 for ^2^H_3,3′_-lactate. Non-interpolated images for this set are presented in Supplementary Figure S2.

Similar DMI analyses were executed on pregnant animals that had been administered l-NAME prior to the MRI scans—a treatment that, as mentioned, mimics preeclamptic conditions. Figure 3 depicts representative results obtained upon injecting a ^2^H_6,6′_-glucose bolus into a pregnant mouse that underwent such treatment. Supplementary Figures S15–S23 show additional ^2^H MRSI images from the *n* = 9 animals that were treated with l-NAME and scanned in a similar manner. (Representative single-voxel NMR spectra extracted from ^2^H MRSI measurements on an animal that had been administered l-NAME and undergone a ^2^H_6,6′_-glucose bolus injection, are shown in Figure 1). Qualitatively speaking, the results on these preeclamptic animals are akin to those in normal mice, with ^2^H_6,6′_-glucose perfusing both placentas and maternal kidneys, and subsequent ^2^H signals arising in these organs for deuterated water and lactate. It once again appears that in certain cases, the perfusion of glucose into the fetoplacental units is somewhat delayed, as it maximizes after it does in the maternal kidney—for fetal organs, ca. 15–20 min after injection (see Supplementary Figures S15–S23 for the actual details). ^2^H_3,3′_-lactate is then generated in the fetoplacental units, reaching maximum levels in the 1–5 mM range ca. 45–90 min after injection. Not only are these levels higher than in the control pregnancies; they appear in additional fetal organs, and seem to last longer than in the former cases. The ^2^H signal from ^2^H-water also increases steadily throughout the course of the experiment (Figure 3B,E and Supplementary Figures S15–S23), both on maternal kidney and placentas as well as in fetal brains, hearts and livers.

**Figure 4:**
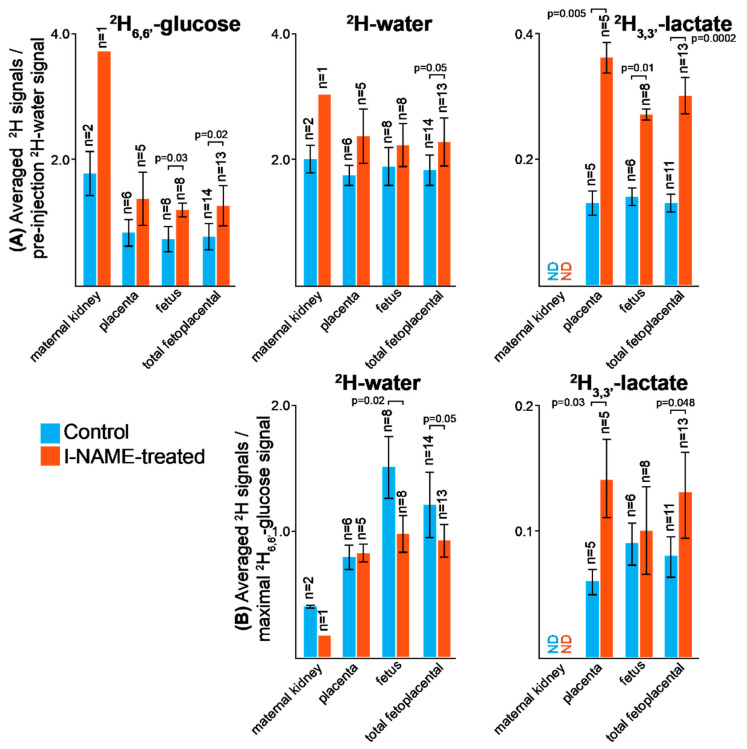
Summary and statistics for the uptake and the generation of various metabolites in maternal and fetal organs, highlighting salient aspects of the experiments run in this study (Supplementary Figures S3–S11 and S15–S23). Both panels focus on glucose, water or lactate signals averaged over the entire time course of the experiment, i.e., if 10 images were taken over a 125 min experiment, what is shown are the sum of the corresponding peak images as mapped for each organ, divided by 10. (**A**) Average signals normalized according to the image intensity observed in each organ for the pre-injection ^2^H-water peak, for the control and l-NAME-treated animals. This gives an idea of the maximum absolute levels reached by each metabolite in each organ. (**B**) Idem as (**A**) but upon normalizing the water and lactate ^2^H images by the maximum ^2^H_6,6′_-glucose signal observed in the animals’ organs. This conveys an idea of glycolytic activity, independent of the glucose injection details. Error bars indicate standard deviations of the measurements for the indicated regions; notice that because of limited spatial resolution, all the fetal organs (brain, liver, heart) are here pooled in the “fetus” category. Indicated as well are *p*-values calculated from paired, two sample *t*-tests assuming unequal variances, distinguishing uptake/activity in control vs. preeclamptic mice for the various metabolites; only significant differences (*p* ≤ 0.05) are noted. ND: non-detectable.

Figure 4 further summarizes the differences and similarities observed by DMI for the control and preeclamptic pregnant mice. For clarity this figure focuses on the average signal intensities retrieved from the various metabolites, upon considering the time-series of ^2^H MRSI acquisitions as a whole. While forgoing kinetic insight, this kind of “boxcar averaging” helps to stress metabolic differences and similarities between the different animal models. Additionally, pooled together in this figure are the various fetal organs (brain, heart, liver), which although often apparent in the images are sometimes hard to resolve by the DMI. Statistics are here presented in two complementary formats. Shown in Figure 4A are averaged signal intensities observed for specific organs over the entire time series, upon normalizing the images by the corresponding pre-injection ^2^H-water signal. This evidences the uptake and generation of glucose’s metabolic products, in terms of their absolute concentrations. In order to decouple overall concentrations from biases that might arise from differences in the glucose’s initial injection or subsequent uptake, which as mentioned may vary somewhat between animals and between fetoplacental units, Figure 4B presents similar time-averaged metabolic intensities—but after these have been normalized by the maximum ^2^H_6,6′_-glucose signal observed for the images of each of the injected animals. This representation thus highlights differences in the rates of glucose conversion, rather than in potential glucose perfusion differences. It follows from the absolute concentration format that whereas it is hard to establish differences in the rates of ^2^H-water formation between the animal models, systematically higher ^2^H_3,3′_-lactate levels are observed for the fetoplacental compartments of the preeclamptic animals. Such trends are in fact visible from a qualitative inspection of the ^2^H MRSI data themselves (Supplementary Figures S3–S23), which yield systematically higher intensities in the ^2^H_3,3′_-lactate images of the l-NAME-treated animals than for the controls. Further analyses show that although after normalization by the glucose level the generation of lactate in fetal organs ends up similar in all animals, lactate’s accumulation in placentas is systematically higher in the preeclamptic animals.

### 2.3. Kinetic Analyses of the DMI Data

**Figure 5:**
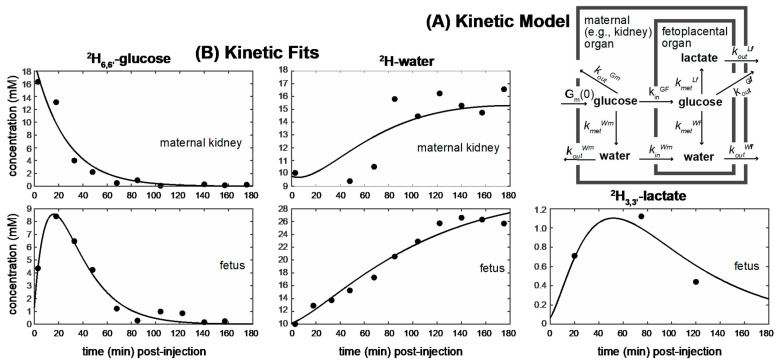
(**A**) Model assumed in this study to describe the exchanges between fetoplacental and maternal glucose, water and lactate, and fit the time evolutions observed for these metabolites by DMI. Indicated are the kinetic constants introduced in the fits (Equations (1)–(5)); missing arrows represent slower or ill-defined processes whose effects were not accounted for in this model; *k_out_*s include kinetic process as well as other generic sources of MR signal loss. Notice that the fitting process also requires an initial ^2^H_6,6′_-glucose concentration *G_m_*(0). (**B**) Example of fits arising from this model for maternal kidney and fetal voxels, observed following an injection of ^2^H_6,6′_-glucose to a pregnant mouse that has been administered l-NAME. Supplementary Figure S25 shows similar quality fits arising for all the data sets analyzed in this study. Average results of these fits are presented in Table S1 for the control and preeclamptic cohorts.

In addition to the time-averaged assessment presented in Figure 4, kinetic time traces were analyzed for the full set of ^2^H MRSI data collected over the whole animal cohorts making part of this study. Given the limitations of the collected ^2^H MRSI data—foremost among these spectral sensitivity limitations that only allowed us to identify water and lactate as glucose’s metabolic products and spatial resolution limitations that limited our assessment of individual fetal organ—a simplified kinetic model was adopted. This model is akin to that usually employed to analyze metabolism in animal pregnancies utilizing radioactive tracers [55,56], and it considers (i) glucose (*G*) → lactate (*L*) and glucose (*G*) → water (*W*) as the only two metabolic processes, and (ii) exchanges solely among two different compartments: one (*m*) describing concentrations in the maternal circulation (as measured for instance in the maternal kidney), and the other (*f*) depicting metabolic concentrations in fetoplacental units including the placentas, fetal brains, etc. (Figure 5A). Notice that since no lactate was ever detected outside the fetoplacental unit, solely glucose and water were assumed to exchange between the maternal circulation pool (*G_m_*, *W_m_*) and the fetoplacental (*G_f_*, *W_f_*) organs; all the lactate was thus assumed to be locally produced in the latter. Notice as well that since over the analyzed timescales glucose is surely metabolized onto other unseen products besides water and lactate, it is not possible to equate the former’s rate of disappearance to the latter’s rates of formation. We also assumed that, given the reduced metabolic pools of the fetoplacental vs. the maternal compartments, the contributions to the maternal signals arising from metabolites perfusing out of the fetoplacental units was negligible—while the reverse is of course not true. The differential equations that summarize the ensuing simplified kinetics (Figure 5A) are, therefore,

(1) $$\frac{dG_{m}}{dt}=-k_{out}^{Gm}\cdot G_{m}$$

(2) $$\frac{dG_{f}}{dt}=-k_{out}^{Gf}\cdot G_{f}+k_{in}^{Gf}\cdot G_{m}\;$$

(3) $$\;\frac{dW_{m}}{dt}=-k_{out}^{Wm}\cdot W_{m}+k_{met}^{Wm}\cdot G_{m}$$

(4) $$\frac{dW_{f}}{dt}=-k_{out}^{Wf}\cdot W_{f}+k_{in}^{Wm}\cdot W_{m}+k_{met}^{Wf}\cdot G_{f}$$

(5) $$\frac{dL_{f}}{dt}=-k_{out}^{Lf}\cdot L_{f}+k_{met}^{Lf}\cdot G_{f}$$

Here, *G_m_*, *W_m_*, *G_f_*, *W_f_*, *L_f_* are time-dependent functions that describe the intensities observed for the various peaks (glucose, water, lactate) in the maternal kidney or fetoplacental organs, and $k_{out}^{X}$, $k_{in}^{X}\;\mathrm{and}\;k_{met}^{X}$ are, respectively, constants describing the signal decay (due to combined outflow and metabolic effects), the inflow, and the metabolic generation, of analyte X. Analytical solutions to these differential equations are presented in the Supplementary Figure S5. Using these solutions to fit the experimental data also demands setting the initial conditions of the experiment, which were assumed to include *W_m_*(0) = *W_f_*(0) = 10 mM, an initial *G_m_*(0) = *G_o_* maternal glucose concentration that was obtained by fitting, and *G_f_*(0) = *L_f_*(0) = 0. Figure 5B presents representative fits depicting the kinetic metabolic traces arising for all species for a pregnant animal that had been administered l-NAME, focusing on the time course of the maternal kidney and on the fetus. Supplementary Figure S26 summarizes similar multi-metabolic fits, for the rest of the cohort/organs investigated in this study. Notice that due to a limited sensitivity arising from a relative low abundance of lactate in the fetoplacental units of the control animals, fits of these data could be obtained mostly for averaged time series where multiple ^2^H_3,3′_-lactate MRSI images were co-added together. The main average rate constants and (maximum) levels reached by *G_m_*(0) arising from these fits for different organs are summarized in Supplementary Table S1, for the full cohort of control and preeclamptic animals that were scanned. A main conclusion that can be gathered from this analysis is that, while respecting the fact that a higher lactate concentration builds up for the fetoplacental organs of the l-NAME-treated animals than for their control counterparts, the fits do not predict statistically significant differences between the glucose → lactate metabolic conversion rates among the two classes of animals. Instead, they ascribe the stronger lactate signals in the preeclamptic animals to a combination of higher glucose in-flow rates, and of slower lactate out-flow ones; these two biases end up building up higher amounts of lactate in the preeclamptic fetoplacental organs than in the control ones. Additionally, worth noticing is the fact that all maximal glucose concentrations end up reasonably scattered in the 20–30 mM range and; these values are close to those arising upon fitting *G_m_*(0) for the maternal kidneys.

### 2.4. Ancillary ^2^H_2,2′_-Glycine Experiments

An intriguing component of these results and of their subsequent modeling relates to the meaning of the water signal. On one hand, the ^2^H MRSI consistently shows a high concentration of the ^2^H-water signal in the fetoplacental areas; this could be an indicator of an active local metabolism, as water is expected as end product of most biological pathways. On the other it is reasonable to assume that since water is highly mobile and extensively perfused, it will be delocalized by circulation throughout the pregnant body. It is thus unclear, overall, to which extent the ^2^H-water signal can be linked to metabolism—particularly over the long timescales over which the protocol is run—and to which extent it reflects circulation. To further clarify this, a pregnant control animal was injected with a bolus of ^2^H_2,2′_-glycine, and studied under conditions similar to those used in the glucose DMI experiments. In the resulting non-localized ^2^H MRS spectra, the animals did not evidence any signal other than those of glycine and water. Further, the time-dependence of this MRS series was similar as that detected in vivo for the glucose injection, with the ^2^H_2,2′_-glycine signal at 3.5 ppm initially peaking at 1–9 min, and eventually enhancing the ^2^H-water resonance over the course of 120 min (spectra not shown). Additionally, as described for glucose, the ^2^H_2,2′_-glycine readily perfused into the maternal kidney and from there entered the maternal circulation (Supplementary Figure S24). However, by contrast to glucose, only a minor fraction of the injected glycine seems to cross the placental barrier and localize in the fetuses: images showed [^2^H_2,2′_-glycine] reaching up to ~10 mM in maternal kidneys, vs. ~0.9 mM in the fetal organs. We conclude from this that the amount of labelled material in fetal circulation that may serve as a substrate for fetal metabolism, is significantly lower in this case than in the ^2^H_6,6′_-glucose injections. Despite this difference, a significant amount of water is detected in these experiments in the fetuses—with [^2^H-water] up to ~25 mM. This indicates that, at least to some extent, the ^2^H-water detected in the fetuses originates via perfusion from the maternal circulation. Still, whereas in the ^2^H_6,6′_-glucose injections the HDO levels detected in the fetuses usually exceed by ~2-fold the levels detected in the maternal kidneys (Figure 4A), in the ^2^H_2,2′_-glycine injections the ^2^H-water levels reached by these two organs, were similar. Assuming then that the fetal ^2^H-water signal observed in the ^2^H_2,2′_-glycine-injection experiments originates mostly from circulation, this suggests that ca. half of the ^2^H-water signatures detected in fetal organs in glucose-based DMI arose from local metabolic processes. Further assessment of how the ^2^H in glucose ends up in water, could open valuable routes to investigating metabolic pathways other than glycolysis [57].

## 3. Discussion

Joint measurements of placental and fetal transport and metabolism can add valuable insight in understanding and managing pregnancies. DMI opens an opportunity for non-invasively mapping these properties, and could serve as complement to the anatomical, functional and diffusivity insights that arise from MRI, ultrasound and other imaging modalities. This potential was here explored by following in vivo the fate of ^2^H_6,6′_-glucose, chosen because of its essential role in fetal nutrition. Injections followed by ^2^H MRSI acquisitions at 15.2T allowed us to image the perfusion of deuterated glucose, which showed up in maternal kidneys and placentas within ~10 min, and on downstream fetal organs a few minutes later. Glucose’s perfusion into healthy mice placentas has also been recently assessed using glucoCEST MRI, where ^1^H-glucose was observed to peak ca. 18 min after injection [38]; however, further glucose perfusion into fetal organs and/or its conversion to other metabolites, could not be assessed with such methodology. By contrast, signatures for both glucose → lactate and glucose → water transformations in maternal, placental and fetal organs, are clearly revealed by ^2^H MRSI. According to these ^2^H measurements, lactate levels are always negligible in the maternal organs (e.g., kidney) but peak in the fetoplacental units of control animals to ~0.5–2 mM, washing out roughly 90 min after injection. In addition, a main difference evidenced by these studies was a consistent, statistically higher lactate level upon glucose injection in the preeclamptic animals than in the controls, both in the placentas and the fetal organs (Figure 4).

The role of glucose availability and of glycolysis in dam, placental and fetal organs, has been a topic of extensive investigations in both animals and humans [58,59,60,61]. The glucose used by fetuses arrives nearly exclusively via placental transport, and its fate has been linked to a pregnancy’s outcome and subsequent neonatal development [62,63,64]. Literature data suggest that fetuses subject to intrauterine restrictions are often hypoglycemic, a trait which has been associated with the ensuing small neonatal sizes and other post-delivery complications. As these hypoglycemic traits are not echoed at the maternal level, this has raised the question of whether fetal hypoglycemia is related to reduced placental transport and/or to increased placental glucose consumption. An elevated glucose metabolism has indeed been found in human placentas of preeclampsia cases [65]; this has been associated with the hypoxic nature associated with this condition, leading to an enhanced need for anaerobic glycolysis. Additionally, other enzymes associated with glycolysis reportedly increase their activity upon exposing placentas to reactive oxygen species, of the kind arising under fetoplacental hypoxia [66]. Studies have also reported an increase in lactate-dehydrogenase and in lactate levels for placentas and fetuses which suffer from growth restrictions [58,59,60,61]. Overall, therefore, the increase in lactate detected by DMI upon injecting ^2^H_6,6′_-glucose into l-NAME-treated pregnant mice, agrees well with these studies associating an increased placental glucose consumption and ensuing lactate generation, with pregnancy complications.

It is interesting to compare these results, with those that we have recently observed using similar preeclamptic rodent models by diffusion-weighted imaging, gadolinium-contrast-enhanced imaging, and hyperpolarized metabolic ^13^C MRSI [9,31,54]. When administering Gd-DTPA to l-NAME-treated pregnant mice, dynamic T_1_ analyses focusing on the initial 100–200 s post-injection, showed a diminished perfusion into the placentas; similar effects arose when these measurements were executed in knock-out mice models of intrauterine growth restriction [54]. Systematic differences between l-NAME-treated or knock-out animals and control pregnant mice, were also observed when characterizing placental diffusivities: MRI measurements then showed systematically lower averages for water’s mean apparent diffusivities in the diseased animal placentas, than in their controls [54]. As for hyperpolarized ^13^C MRSI: these studies detected no meaningful drop (in fact a statistically inconclusive increase) in the overall amount of injected pyruvate going into the placentas, yet a substantial drop in the rates of ^13^C_1_-pyruvate → ^13^C_1_-lactate placental conversions for the preeclamptic animals [31]. At first sight, the decreased rates of perfusion and diffusion observed by ^1^H MRI and the lower rates of pyruvate → lactate conversion observed by hyperpolarized ^13^C MRSI in the preeclamptic animals, may appear to contradict the picture emerging from the present DMI work. In effect, according to the ^2^H MR images presented in Supplementary Figures S3–S23, lactate generation is in fact enhanced in the fetoplacental units of l-NAME-treated animals. This, however, may be a reflection of the very different timescales and the different physiological processes probed by each experiment. The Gd-enhanced ^1^H dynamic contrast measurements and the pyruvate → lactate conversion process observed by hyperpolarized ^13^C MRSI are heavily biased towards the short-term perfusive behavior of molecules into and through the placentas; so are diffusion-weighted measurements, reporting on apparent water mobility on 10s-of-ms timescales. These active perfusive measurements appear to be impaired in the l-NAME-treated animals. Furthermore, if as derived from other literature studies the concentration of “cold” lactate is indeed higher in preeclamptic than normal placentas, it is liable to assume that the pyruvate ⇔ lactate equilibrium controlled by lactate dehydrogenase and by various transporters, will be biased against further processing of hyperpolarized ^13^C_1_-pyruvate into hyperpolarized ^13^C_1_-lactate. This would further decrease the ^13^C_1_-pyruvate → ^13^C_1_-lactate production rates observed in the preeclamptic rats [31]. By contrast, all of these factors will weight much less in the protracted, hour-long timescales that DMI uses to evaluate the glucose → lactate transformation.

Rates for the glucose → lactate process have been quantitatively measured in a number of studies, particularly on normal and diseased large animal models where a variety of carbon- and tritium-based radioactive tracers (glucose, lactate, water) were injected into the maternal circulation, and their products examined in the maternal and fetal blood and plasma streams [55,56]. Of particular relevance to this DMI study are reports on the rates of ^14^C/^3^H-glucose consumption and ensuing fetal ^14^C/^3^H-lactate generation, as these constants are akin to the $k_{met}^{Lf}$ used in Figure 5A’s model. Healthy ovine and lamb fetal studies report turnover rates of 0.002 and 0.008 ± 0.001 mmol/kg/min for this conversion, respectively; these figures compare well with those independently extracted from the fits in Table S1, which are in the (0.007–0.008) ± 0.002 mmol/kg/min range for the control and preeclamptic mice.

In addition to the formation of a putative lactate peak, the injection of ^2^H_6,6′_-glucose leads to of a very clear ^2^H-water signature. This signal starts as a ~10 mM natural abundance background, and increases several-fold throughout the time course of the post-injection experiment in both control and preeclamptic animals. This excess HDO will at least partially originate via the Krebs cycle [41]. Given the long times involved in our DMI studies one would not normally rely on water to localize metabolism; instead, one would expect HDO to provide a fuzzy picture, as it washes-in and -out of organs throughout the body. Remarkably, however, the images indicate that the growing ^2^H-water resonance has a persistent localization pattern, that resembles an overlay of the ^2^H_3,3′_-lactate and ^2^H_6,6′_-glucose maps (Supplementary Figures S3–S25). To further elucidate this, preliminary experiments were carried out utilizing deuterated glycine—a metabolite which does not cross the placental barrier to an appreciable extent. The appearance of attenuated fetoplacental HDO signatures in these experiments, lent credence to a mix of local water production and perfusion from other organs. Unfortunately, ancillary ex vivo NMR experiments (not shown) failed to shed much light on this matter. The key physiological steps behind ^2^H-water’s production and its apparent localization in DMI, thus, remain to be discerned.

## 4. Materials and Methods

**Figure 6:**
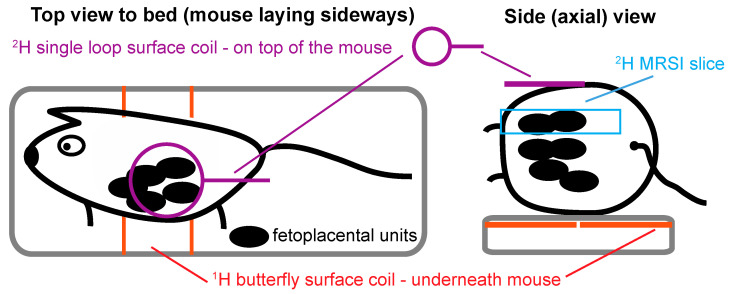
Cartoon depicting the layout used in the experiments described in this study; the ^1^H butterfly coil is embedded in the bed itself.

Animal experiments were approved by the Weizmann Institute IACUC, which is accredited by the AAALAC, the US NIH Office of Laboratory Animal Welfare and the Israel Ministry of Health. In order to carry out the MR experiments, a total of *n* = 26 pregnant Hsd:ICR (CD1) mice (Envigo, Jerusalem) in days E13.5–E19.5 of gestation were initially anesthetized with 3% isoflurane at 20% O_2_ and 80% N_2_, and the levels of isoflurane subsequently lowered to ≈1–1.5% throughout the experiment. Mice were catheterized in their tail-veins, and positioned lying sidewise in a Bruker Biospec at 15.2 T MRI scanner running under Paravision 6. A “sandwich” coil setup was used for scanning the animals, whereby a 20 × 45 mm Bruker ^1^H butterfly surface coil tuned to 650 MHz was placed underneath the mouse, and a customized 20 mm single-loop surface coil tuned to ^2^H’s 99.8 MHz Larmor frequency was placed on top of the mouse’s belly. This allowed us to cover sizable, overlapping portions of the mice’s abdominal regions by both ^1^H and ^2^H MRI with relatively good RF homogeneity; Figure 6 presents the layout of the RF coils and typical animal positioning used in this study. Animals were kept at an approximate body temperature of 37 °C by laying them over a water-heated pad. Mice with preeclamptic symptoms were obtained by administration of Nω-Nitro-L-arginine methyl ester hydrochloride (l-NAME, Sigma-Aldrich, Rehovot, Israel). L-NAME is a vasoconstrictor that inhibits nitric oxide synthase, leading to symptoms that recapitulate many of the features observed in human preeclamptic pregnancies [52,53,54]. A dose of 20 mg/kg_body_weight/day of l-NAME in PBS was administered subcutaneously two days before the MRI experiment, for scans performed on *n* = 9.

^1^H anatomical images were collected using multi-slice TurboRARE or FLASH sequences involving 28–38 slices, 0.5 mm slice thickness, and an in-plane resolution of 0.2 mm. FLASH images were acquired with a flip-angle of 20°, echo-time of 2.8 ms, repetition-time of 270 ms. TurboRARE was acquired with a RARE factor of 10, flip-angle of 90°, echo-time of 28 ms, repetition-time of 2800–3200 ms. Before collecting the ^2^H MRS and MRSI data, animals were intravenously administered 99.5% enriched ^2^H_6,6′_-glucose (Cortecnet, Voisins-le-Bretonneux, France) dissolved in PBS, at a dose of 2.3 g/kg body weight. Most of these tests involved a single bolus injection of 0.25 mL within 60 s (*n* = 20); in *n* = 3 cases an infusion-like injection was performed involving the introduction of several 25–50 μL aliquotes of [^2^H_6,6′_-glucose] ≈ 2600 mM over 60–120 min; since no significant metabolic inferences could be made from these measurements (see above), such infusions were discontinued. Non-localized ^2^H MRS data sets were acquired with a flip-angle of ≈20°, an acquisition time of 100 ms, 0 ms recycle delay, and 128 transients (total acquisition time ≈13 s). Spatially resolved ^2^H MRSI data were collected in the absence of motion triggering, using a k-dependent, weighted-average, chemical-shift imaging (CSI) sequence from Paravision 6. This collected 320 signal averages for the center of k-space and progressively less for its periphery, delivering a data set every ~8 min using slice-selective flip-angles of ≈90°, 60 ms acquisition times, 8 × 8 *k*-matrix sizes (subsequently zero-filled to 32 × 32 elements; see Supplementary Figure S1 for a depiction of the effects from zero-filling) sampled on a Cartesian grid, and repetition times TR = 95 ms. Slice thicknesses varied between 4–8 mm and were tuned to ensure they contained several relevant fetoplacental units; given the relatively thick slices used and the limited FOV provided by the ^2^H surface coil, 3D CSI encoding methods were not assayed. In-plane fields-of-view (FOVs) were 45 × 45 mm. To estimate the effective in-plane spatial resolution, point spread functions (PSF) taking into account the FOV and the Hamming k-space averaging mode employed during CSI acquisitions were calculated. From these PSF’s resolution we estimate the in-plane spatial resolution at ≈5 × 5 mm^2^. This means that certain organs such as the placentas or fetal brains could be reliably resolved from single voxel data; smaller organs that in principle showed well localized signal (e.g., fetal hearts), could be affected due to spill-overs from other organs. Because of these constraints, kinetic analyses were constrained to global placental and fetal regions. ^2^H MRSI data were reconstructed in Matlab^®^ (The Mathworks, Natick, MA, USA) using custom written code. Non-localized ^2^H MRS and slice-selective ^2^H MRSI sets were acquired in alternated, interleaved blocks spaced ~15–20 min, starting from before and continuing for ca. 120 min after the deuterated glucose’s injection. 

^2^H T1s were estimated for the various targeted metabolites at 200 mM concentrations using phantoms made of a 2% agarose gel at physiological salt concentration and pH (data not shown); these were 41 ms for ^2^H_6,6′_-glucose, 392 ms for ^2^H-water (Tzamal D-Chem, Petach Tikva, Israel) and 251 ms for ^2^H_3,3′,3′’_-lactate (Cambridge Isotopes, Tewksbury, MA, USA), as determined by saturation recovery ^2^H MRS. Given these values and the acquisition conditions employed, whereby the targeted slices were chosen parallel to the ^2^H coil and hence were characterized by relatively homogeneous excitation angles, we estimate that when peak intensities were translated into concentrations based on their ratio vs. the natural abundance ^2^H-water observed pre-injection [40,41], glucose concentrations ended up overestimated by ≈10%. As the spatial profile of the surface coil employed coupled to the varying depths of the chosen slices led to similar uncertainties, we decided not to correct for these distortions.

## 5. Conclusions

^2^H MRSI provides a minimally invasive metabolic imaging approach, that was here tested to explore pregnancies. The incorporation of intravenously injected ^2^H_6,6′_-glucose into various maternal and fetoplacental mice organs could be successfully mapped, and its metabolic transformation into water and lactate as resulting from Krebs and glycolytic pathways followed. The in vivo metabolic maps arising from these studies were repeated in pregnant mice models with preeclamptic symptoms, which evidenced substantially higher lactate accumulation than in control counterparts. This is in overall agreement with known features of preeclampsia that suggest higher fetoplacental lactate concentrations; time-traces arising from the DMI data suggest that this does not necessarily reflect higher levels of metabolism, but rather biases in the glucose wash-in and in the lactate wash-out rates.

The glucose dosage used in these DMI studies was 2.3 g/kg body weight. This dose is not much higher than that administered in glucose tolerance tests that look for gestational diabetes (50–100 g per patient) [67,68]. Moreover, our results indicate relatively small differences between the lactate outcomes of bolus injections vs. slower infusions, opening up a route to decreasing the amounts of injected deuterated agents and/or spreading them over longer periods. All this bodes well for future translations of this kind of study into humans.

The present study focused on results obtained upon injection of ^2^H_6,6′_-glucose and ^2^H_2,2′_-glycine. Additional tests based on perdeuterated glucose injection (not shown) gave inferior results in terms of spectral resolution than the ones presented in this study, with relatively broad resonances and overlapping peaks despite the 15.2T field used. Limited spectral resolution is likely to persist as a limitation of this method, as ^2^H’s rapid quadrupolar relaxation will in general be associated with a limited capacity to resolve multiple peaks. This constraint was also apparent by our limitation to pinpoint any metabolic product other than water and lactate, and stands in contrast to ^13^C’s hyperpolarized MRSI ability to routinely discriminate ^13^C_1_-pyruvate from ^13^C_1_-pyruvate-hydrate, ^13^C_1_-alanine, ^13^C_1_-lactate and additional glycolytic intermediates [69,70]. Another noteworthy point about the present in vivo ^2^H studies is their limited sensitivity, which limited in turn the spatial resolution with which the fetal organs could be targeted. Alternative MRSI scanning methods endowed with better sensitivity/unit_time than the chemical shift imaging approach used here can be envisioned [71], while the relatively slow kinetics involved in infusion tests means that longer signal averaging periods and their coupling to various compressed sensing schemes, could also be conceived [72]. We are currently investigating if all these features could combine into making a more powerful methodology for studying pregnancies and their disorders in animals, as well as their potential translation to human-relevant magnetic field strengths.

## Data Availability

All the data that was collected for this study is presented in the Supplementary Materials for evaluation, and is available electronically upon request.

## Supplementary Materials

The following material is available online at https://www.mdpi.com/article/10.3390/metabo11060376/s1. Figures S1 and S2: On the effects of zero filling on the spatial resolution of the ^2^H MRSI images, Figures S3–S11: DMI analyses on control pregnant animals (bolus injections), Figures S12–S14: DMI analyses on control pregnant animals (≈60 min long glucose infusion), Figures S15–S23: DMI analyses on l-NAME-treated animals (bolus injections), Figure S24: Ancillary ^2^H analysis (glycine), Equations (S1)–(S10): Solutions to the kinetic model put forward in Figure 5, Figure S25: Spectrally resolved kinetic ^2^H MR fits for all organs in the control and l-NAME-treated animals analyzed in this study (Figures S3–S12 and S13–S21), Table S1: Average kinetic constants determined for the control and l-NAME-treated mice derived from the kinetic analyses presented in Supplementary Figure S25.
