# Supplementary material for: Deuterium Magnetic Resonance Imaging and the Discrimination of Fetoplacental Metabolism in Normal and L-NAME-Induced Preeclamptic Mice
Source: Metabolites. 2021 Jun 10;11(6):376. doi: 10.3390/metabo11060376 (PMC8230481; doi:10.3390/metabo11060376)
Supplement: Supplementary file 1 [file metabolites-11-00376-s001.zip › metabolites-1208871-supplementary.pdf]

Supporting Information for

Deuterium magnetic resonance imaging and the discrimination of fetoplacental metabolism in normal and l-NAME-induced preeclamptic mice

Stefan Markovic[1], Tangi Roussel[2], Michal Neeman[3] and Lucio Frydman[1,*]

[1]Department of Chemical and Biological Physics, Weizmann Institute of Science, Rehovot, Israel

[2]Center for Magnetic Resonance in Biology and Medicine, Marseille, France

[3]Department of Biological Regulation, Weizmann Institute of Science, Rehovot, Israel

*To whom correspondence should be addressed. E-mail: lucio.frydman@weizmann.ac.il

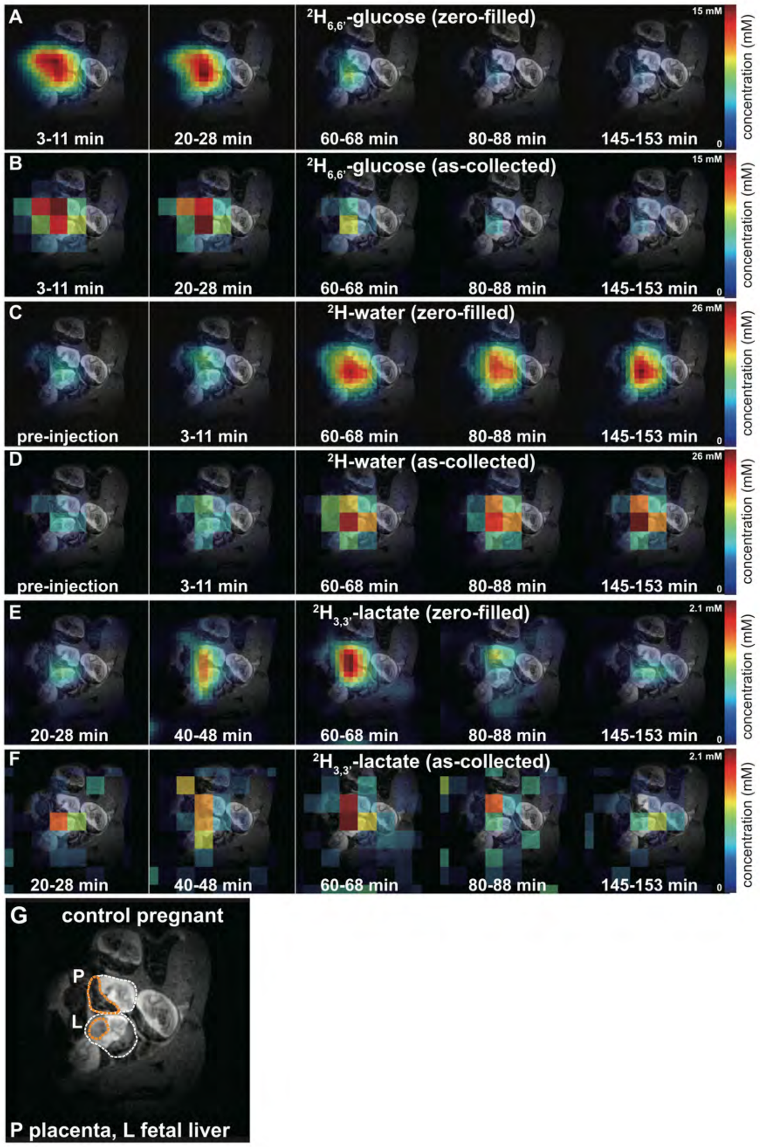

Supporting Information S1. On the effects of zero filling on the ^{2}H MRSI images

The ^{2}H MRSI maps presented in this study were collected using an 8x8 phase-encoding matrix, zero-filled to 32x32 points before Fourier processing. This section presents the effects of such zero-filling procedure, using the ^{2}H images shown in the main text as prototypical examples.

Figure S1. Comparison between the images arising from as-collected (8x8 matrix sizes) data used in this study, and the images arising following zero-filling of the k-space data to 32x32 before FT. Data in this Figure corresponds to the images shown in Figure 2 of the main text, using a similar notation. Also indicated are some of the organs.

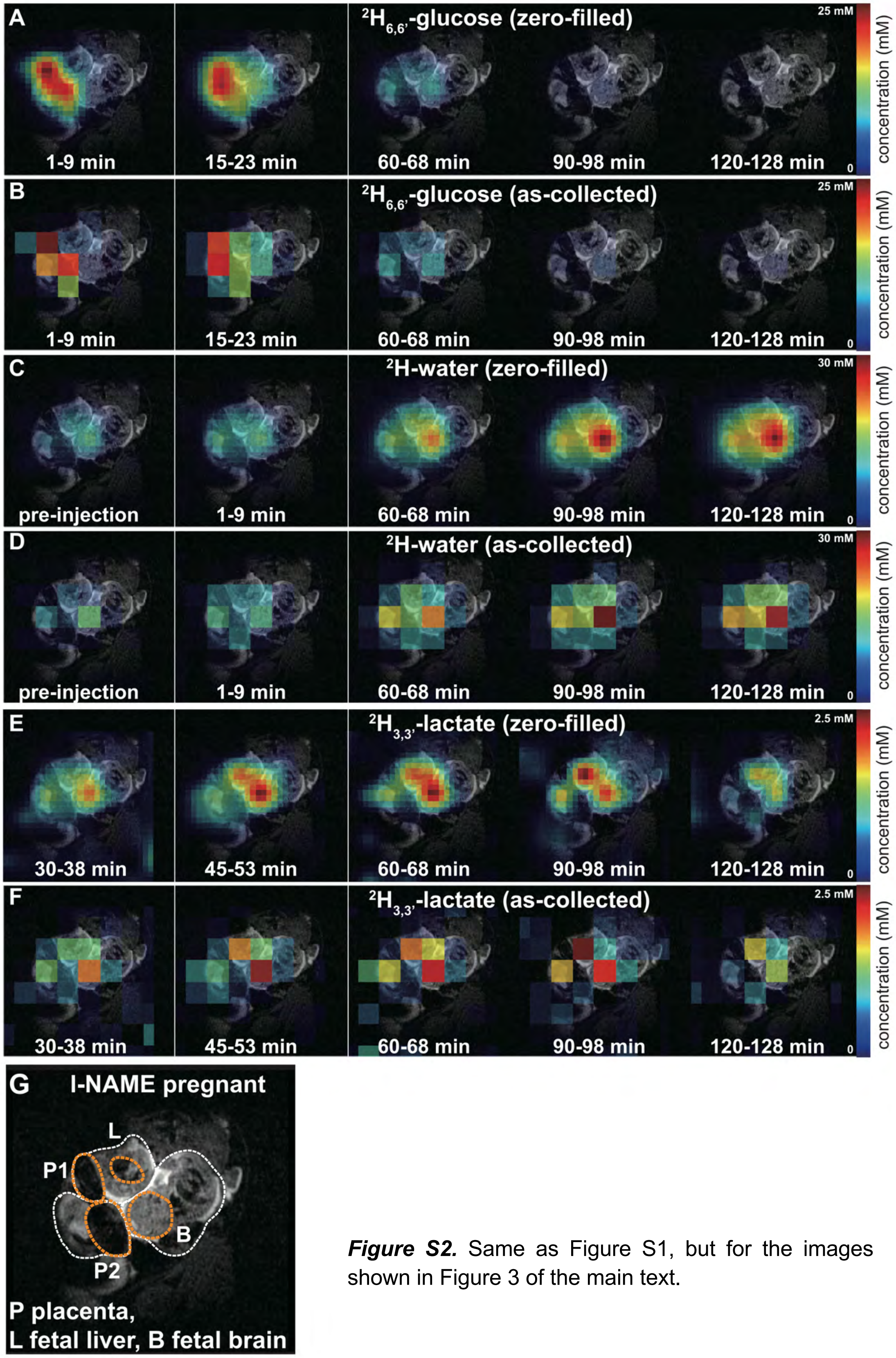

Figure S2. Same as Figure S1, but for the images shown in Figure 3 of the main text.

Supporting Information S2. DMI analyses on control animals (bolus injections)

This section presents the time-series of ^{2}H MRSI maps collected after an intravenous bolus of $^2H_{6,6'}$-glucose was administered to the n=9 pregnant mice used in this study as controls. All ^{2}H MRSI images were recorded as described in the main text over a time course >2 h. In all cases the top rows present an anatomical reference image indicating organs and the time-averaged metabolic maps resulting from the study for $^2H_{6,6'}$-glucose and its metabolic products ^{2}H-water and $^2H_{3,3'}$-lactate. Their colormaps are normalized to the maximum signal (water). Shown underneath these spectra are the series of site-resolved ^{2}H MR images collected in the exam overlaid with anatomical ^{1}H MR references, as well as time traces for organs of interest as identified below the images (as defined within the limits of our spatial resolution).

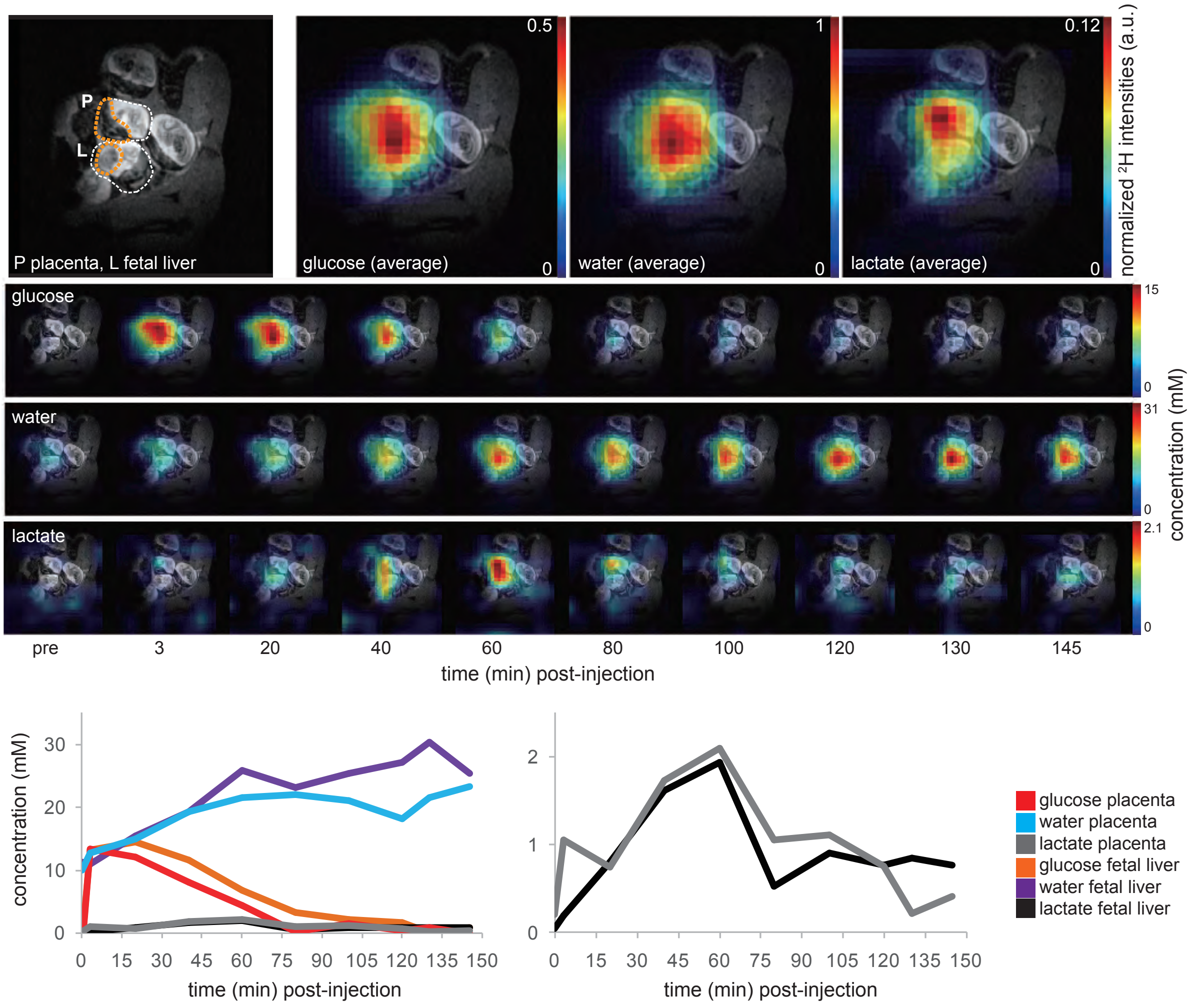

Figure S3: Akin to figure 2 in the main text. Glucose is initially perfusing the placenta with a peak at 3-11 min after injection at 14 mM and the fetal liver with a peak at 20-28 min after injection at 15 mM. Water is steadily produced in the fetal liver rising to 31 mM. Lactate is produced in the placenta right away with a peak at 60-68 min at 2.1 mM and at 60-68 min after injection at 2 mM in the fetus (apparently in the liver). Lactate in both compartments washes out rapidly.

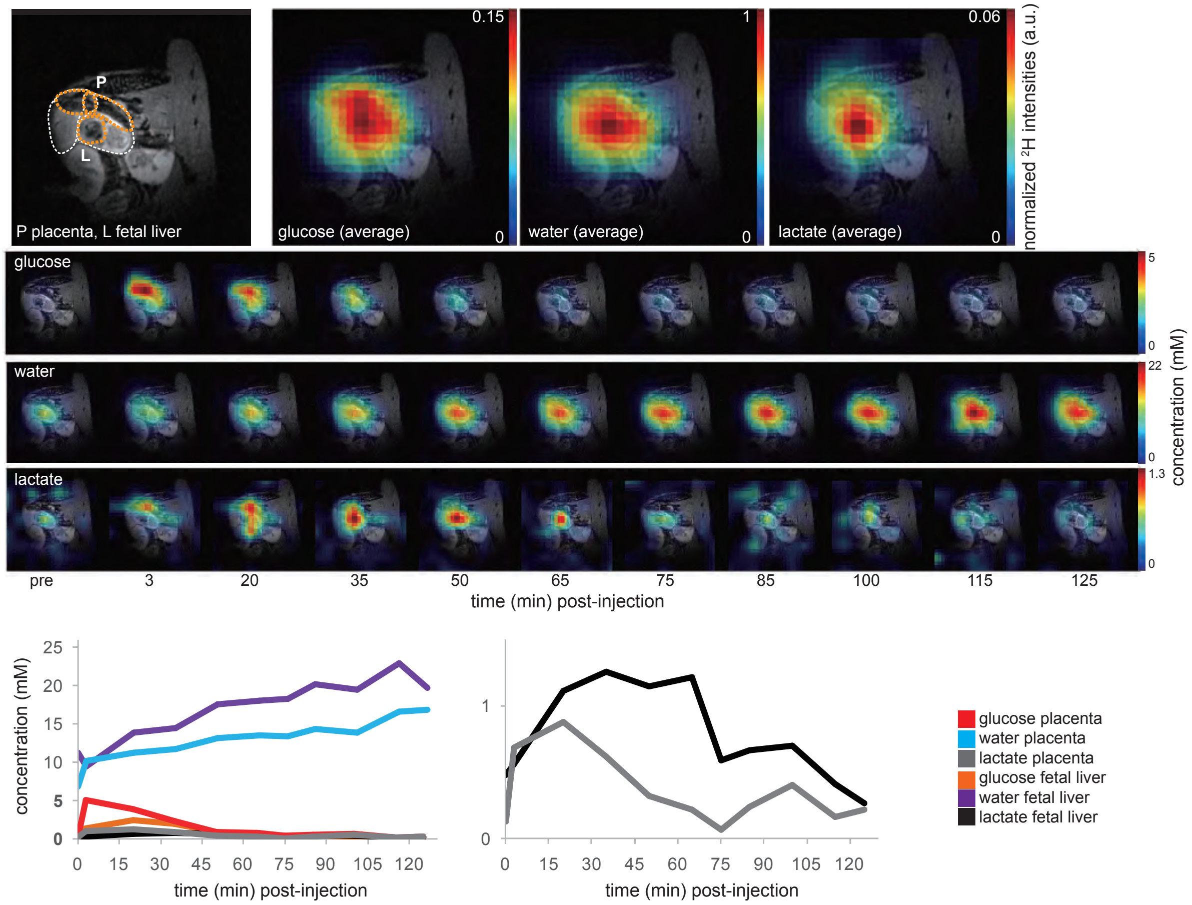

Figure S4: Idem as Figure S3 but on a different animal. Glucose is initially perfusing the placenta with a peak at 3-11 min after injection at 5 mM and the fetal liver with a peak at 20-28 min after injection at 2.5 mM. Water is steadily produced in the fetal liver rising to 22 mM. Lactate is produced in the placenta right away with a peak at 20-28 min at 0.9 mM and at 60 min after injection at 1.3 mM in the fetus (apparently in the liver). Lactate in both compartments washes out rapidly.

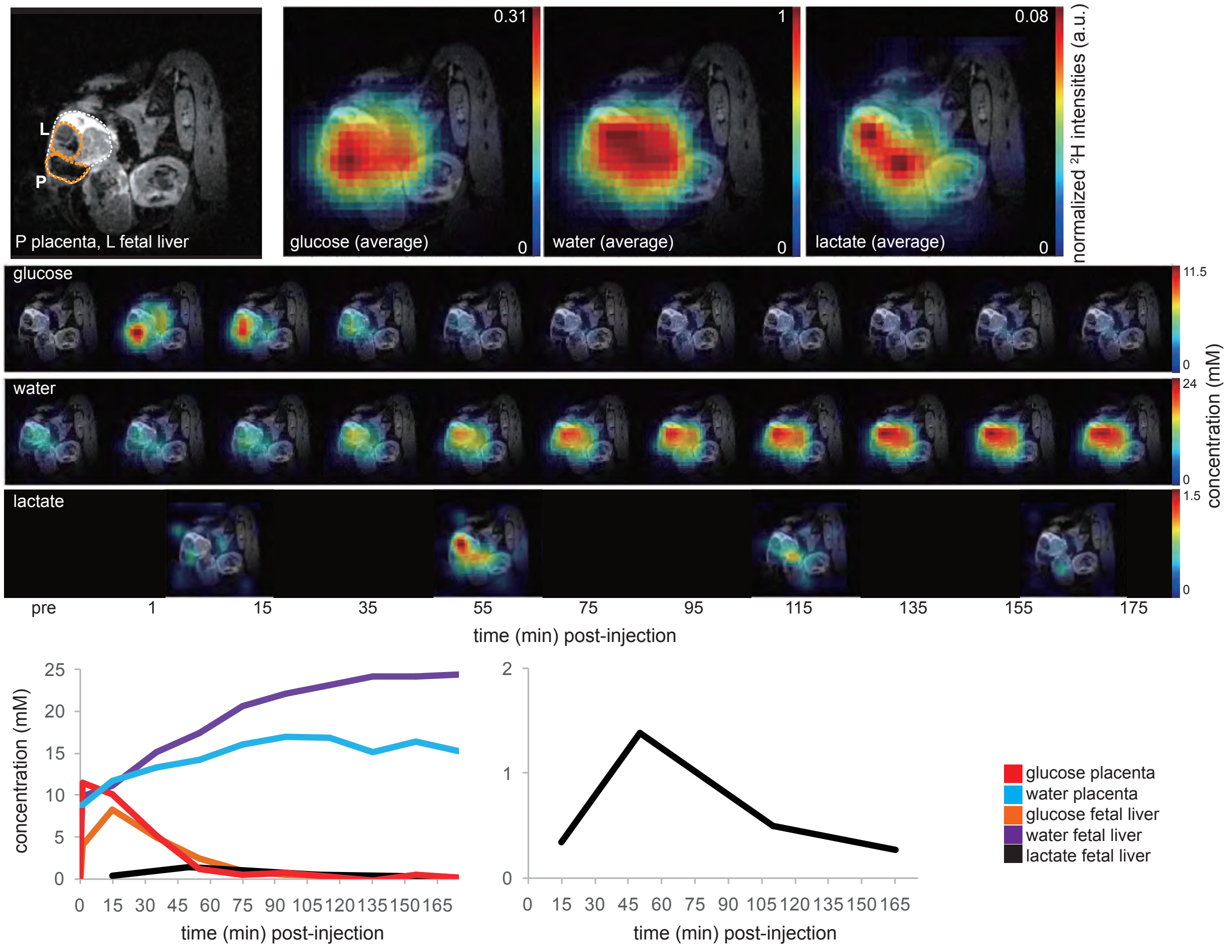

Figure S5: Idem as Figure S3 but on a different animal. Glucose is initially perfusing the placenta with a peak at 1-9 min after injection at 11.5 mM and the fetal liver with a peak at 15-23 min after injection at 8.2 mM. Water is steadily produced in the fetal liver rising to 24 mM. Lactate is produced in the fetus (apparently in the liver) with a peak at 35-38 min at 1.5 mM, and washes out rapidly.

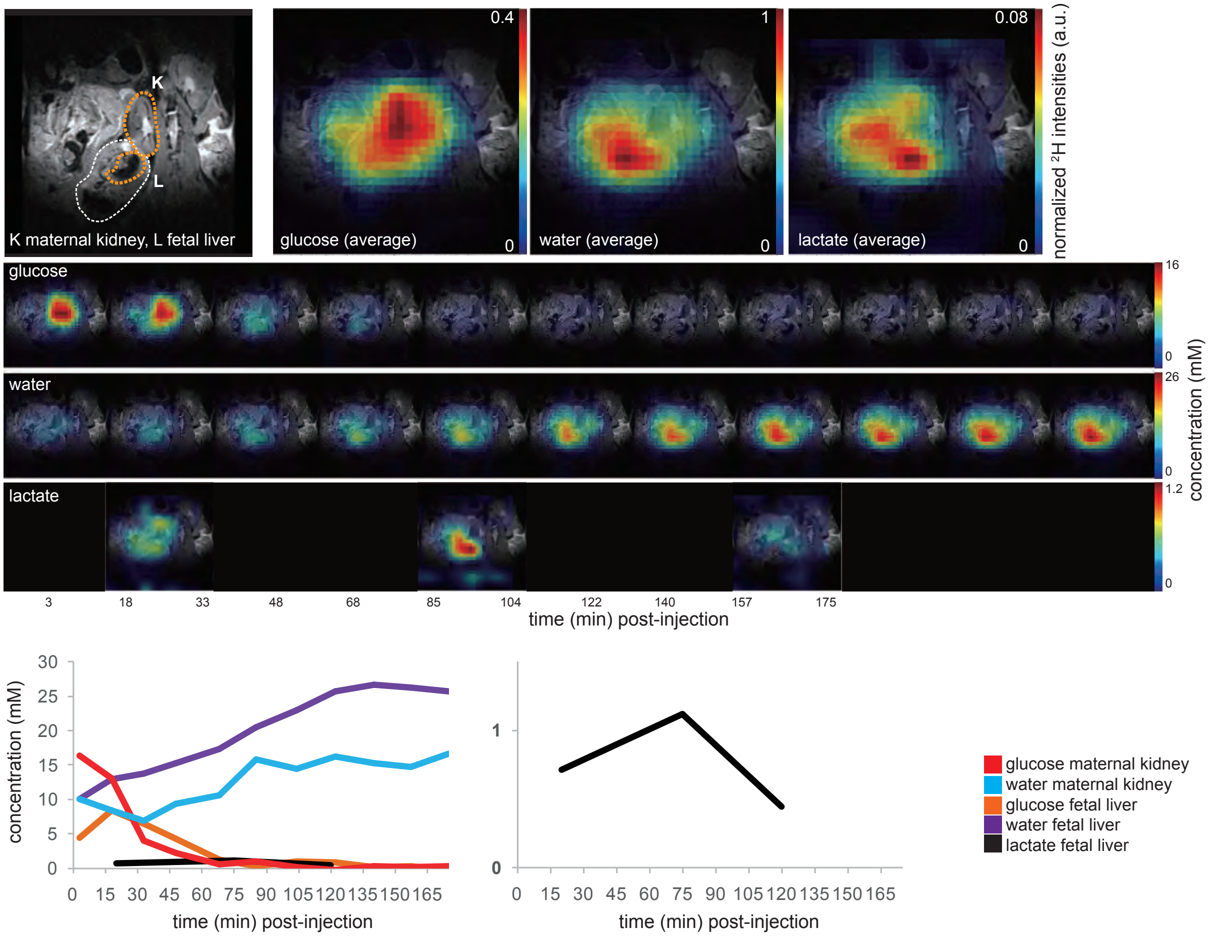

Figure S6: Idem as Figure S3 but on a different animal. Glucose is initially perfusing the maternal kidney with a peak at 3-11 min after injection at 16 mM and the fetal liver with a peak at 18-26 min after injection at 8.4 mM. Water is steadily produced in the fetal liver rising to 26 mM, whereas water production in the maternal kidney is less pronounced at 16 mM. Lactate is produced in the fetal liver with a peak at 48-93 min at 1.2 mM, and washes out rapidly.

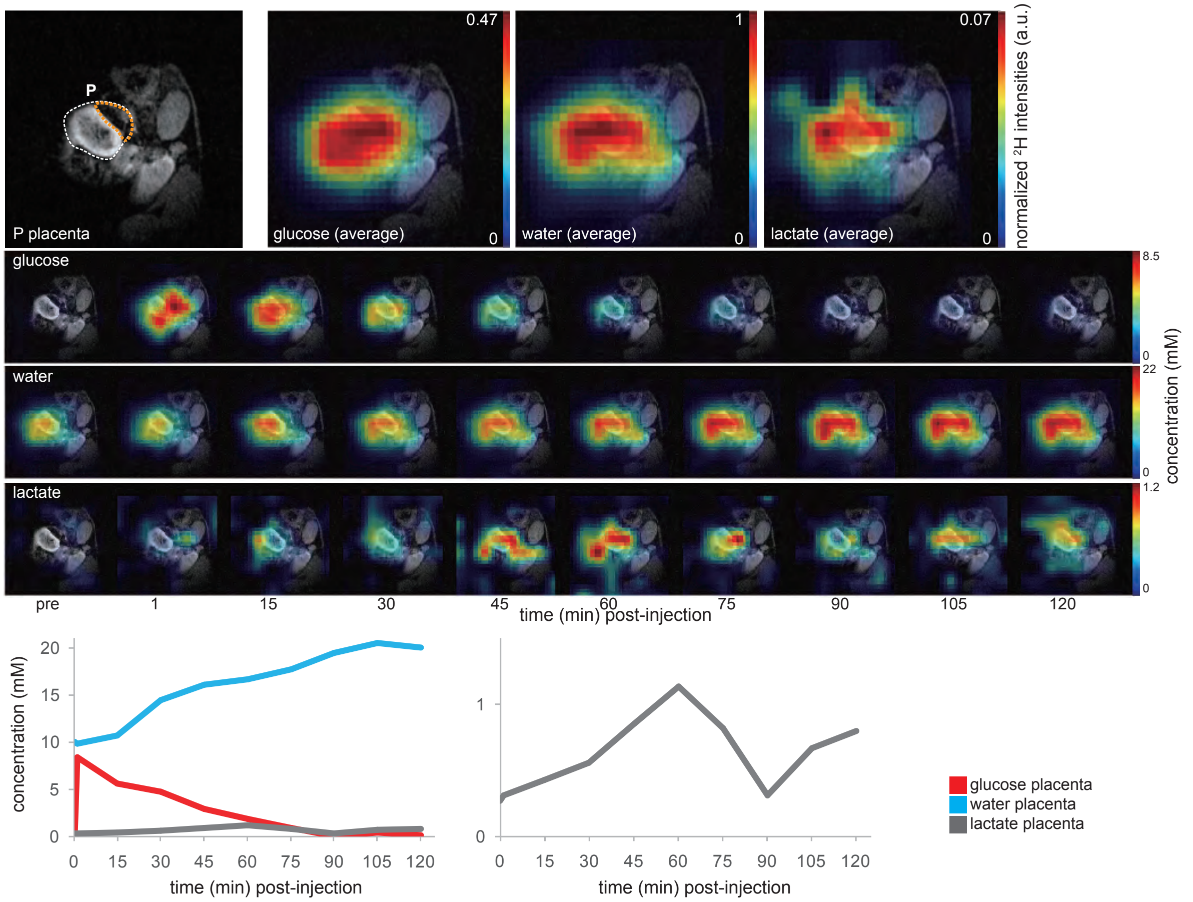

Figure S7: Idem as Figure S3 but on a different animal. Glucose is initially perfusing the placenta with a peak at 1-9 min after injection at 8.5 mM. Water is steadily produced in the fetus (apparently in the liver) and the placenta. In the latter rising to 21 mM. Lactate is produced in the placenta with a peak at 60-68 min at 1.2 mM.

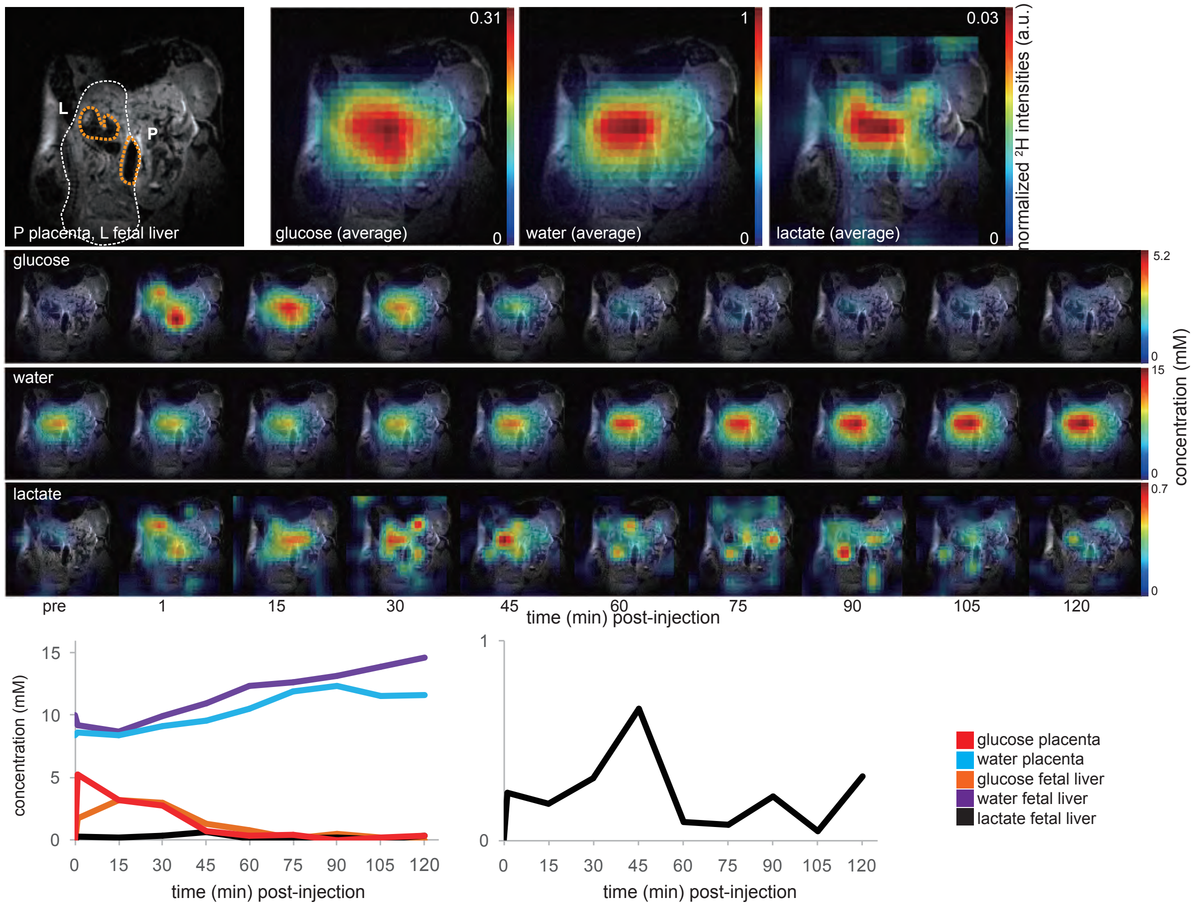

Figure S8: Idem as Figure S3 but on a different animal. Glucose is initially perfusing the placenta with a peak at 1-9 min after injection at 5.2 mM and the fetal liver with a peak at 15-23 min after injection at 3.2 mM. Water is steadily produced in the fetus (apparently in the liver) rising to 15 mM. Lactate is produced in the same location with a peak at ~50 min at 0.7 mM.

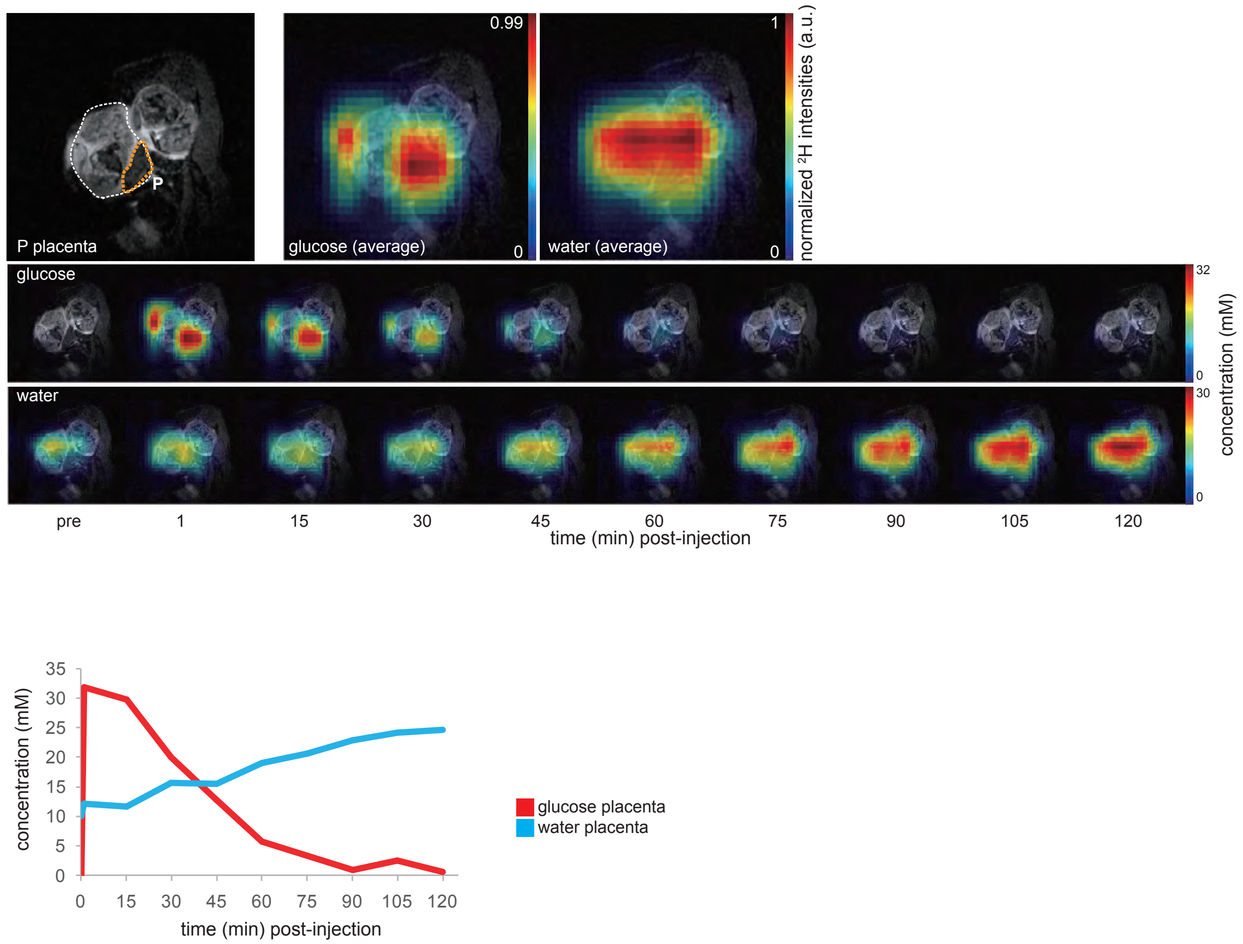

Figure S9: Idem as Figure S3 but on a different animal. Glucose is initially perfusing the placenta with a peak at 1-9 min after injection at 32 mM. Water is steadily produced in the placenta rising to 26 mM. No lactate peak is observed.

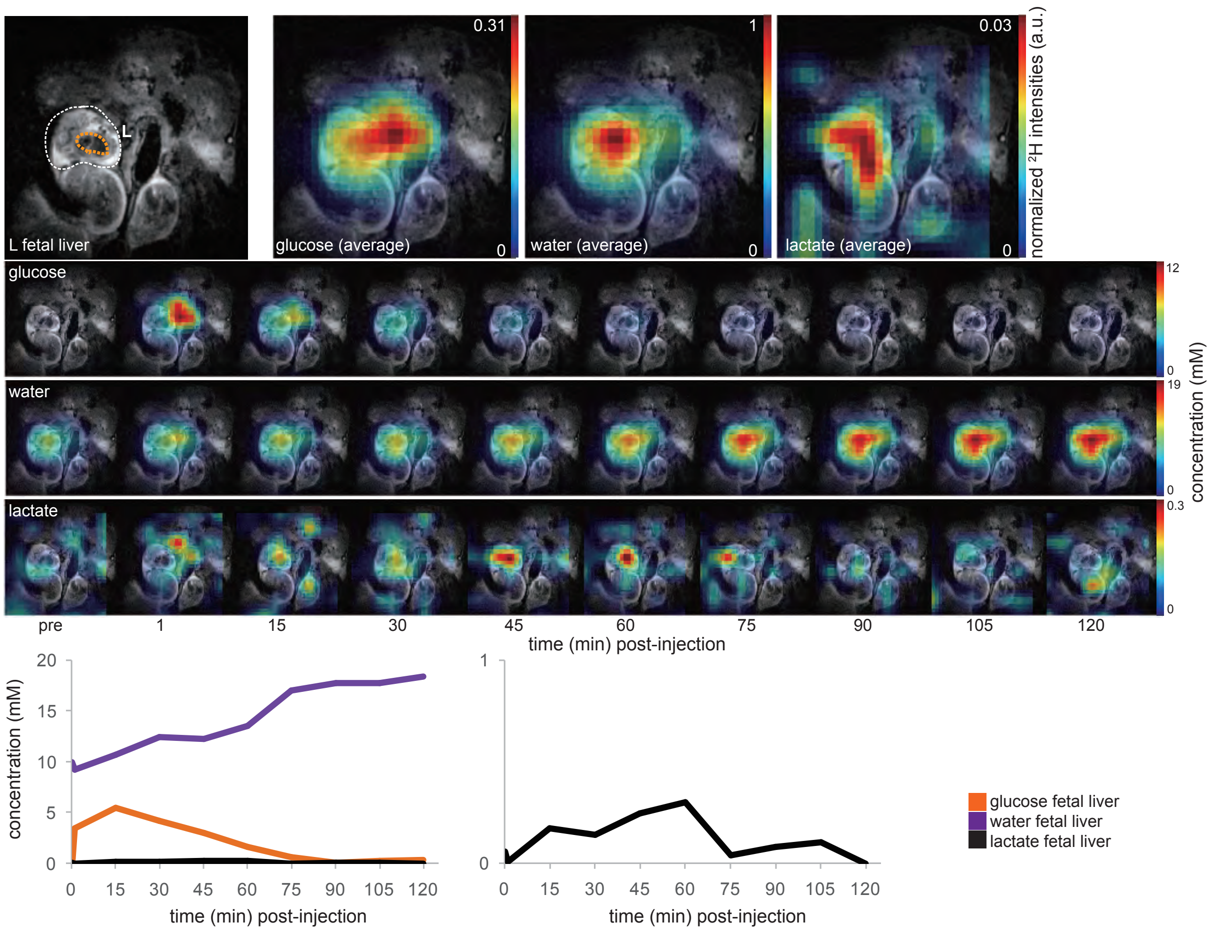

Figure S10: Idem as Figure S3 but on a different animal. Glucose is initially perfusing the fetus with a peak at ~15 min after injection at 5.5 mM. Water is steadily produced in the fetus rising to 19 mM. Lactate is minimally produced in the fetus with a peak at ~60 min at 0.3 mM.

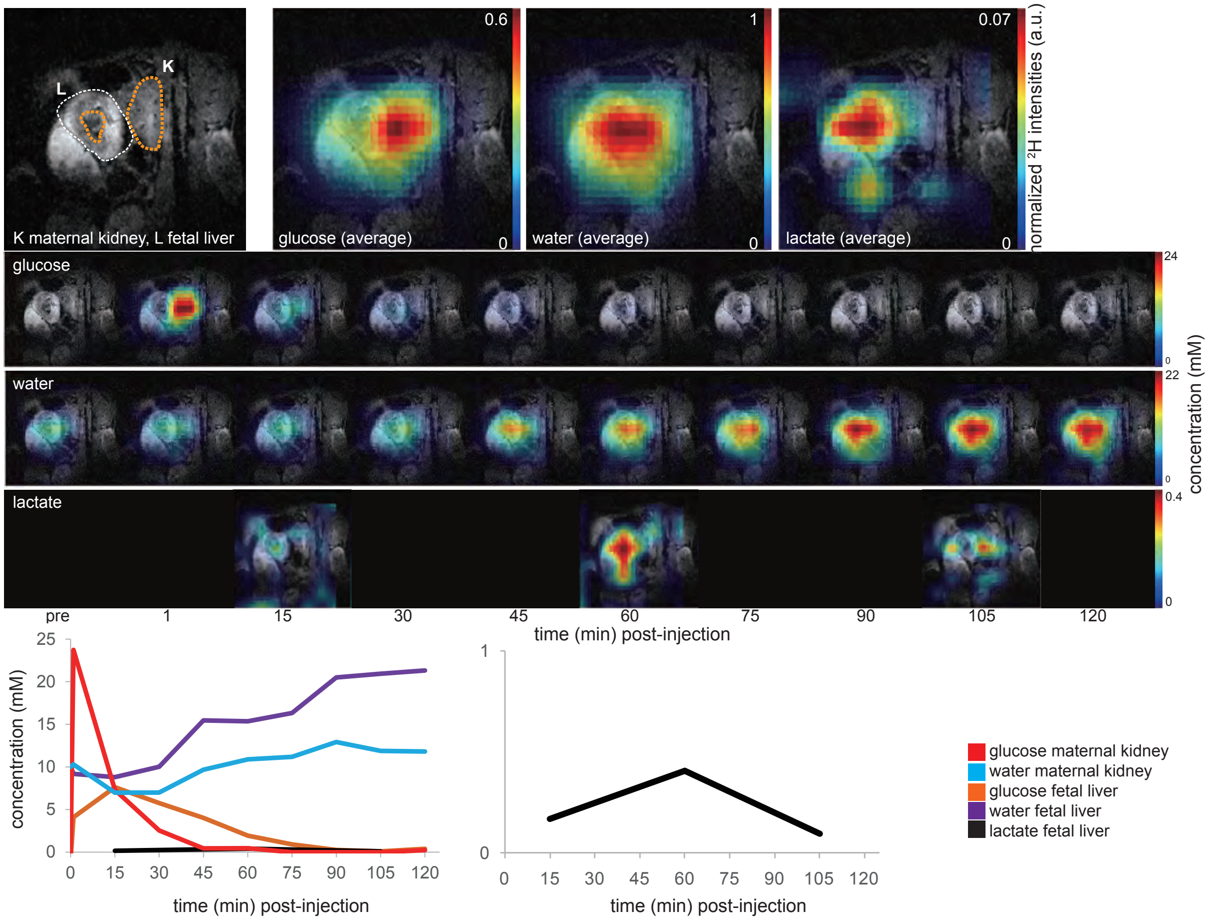

Figure S11: Idem as Figure S3 but on a different animal. Glucose is initially perfusing the maternal kidney with a peak at 5-10 min after injection at 24 mM and the fetal peak at ~20 min after injection at 7.6 mM. Water is steadily produced in the fetus rising to 22 mM, whereas water production in the maternal kidney is less pronounced. Lactate is produced in the fetus (apparently in the liver) with a peak at ~60 min of 0.4 mM.

Supporting Information S3. DMI analyses on control animals (≈60 min long glucose infusion)

This section presents the time-series of ^{2}H MRSI maps collected after slow intravenous infusion of $^2H_{6,6'}$-glucose administered to n=3 pregnant mice as described in the main text; these data sets were not used for the analysis presented in the main text around Figure 4. All other details are as in the preceding section.

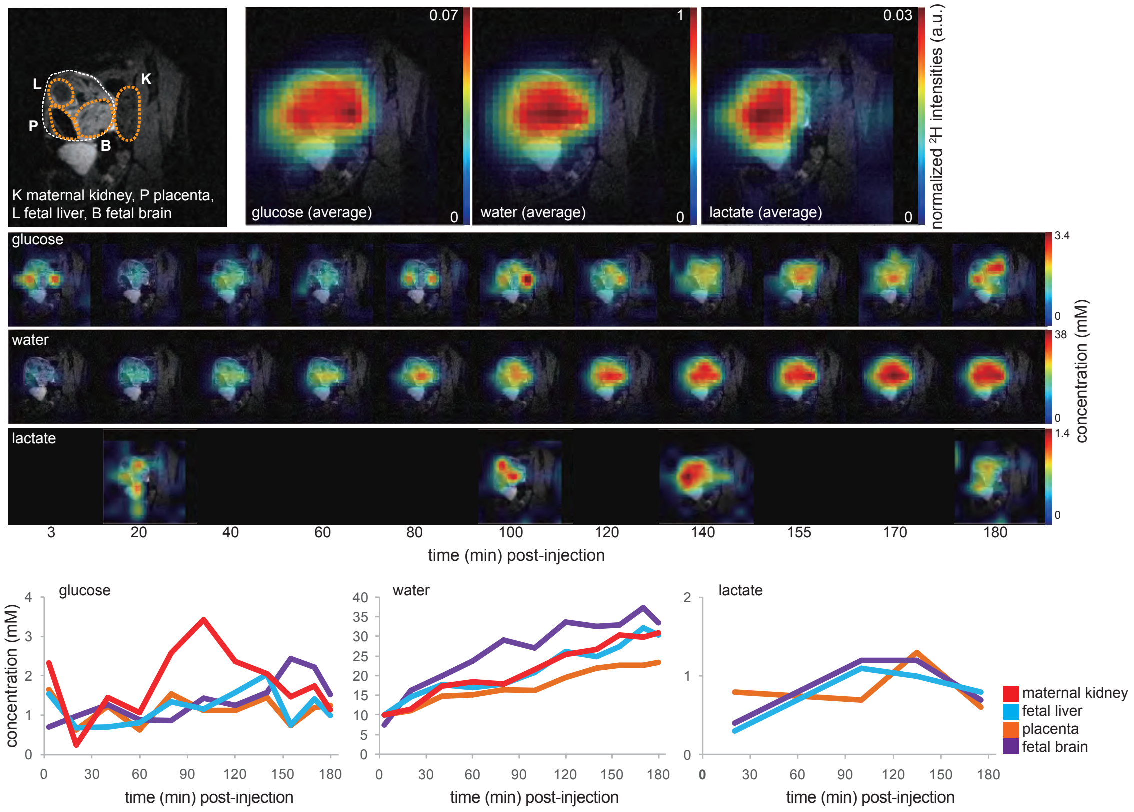

Figure S12: Akin to Figure 2 in main text, but with a control pregnant mouse slowly infused as described in Methods. Glucose initially localizes in the maternal kidney and the placenta, but later on distributes over multiple fetal tissues. Unlike in the bolus injection case glucose concentrations are kept mostly ≤3.4 mM. Water however is steadily produced in all organs rising up to 38 mM as in the bolus injections. Lactate is produced in the placenta and in fetal organs, with a similar concentrations as in the bolus injections and up to 1.4 mM.

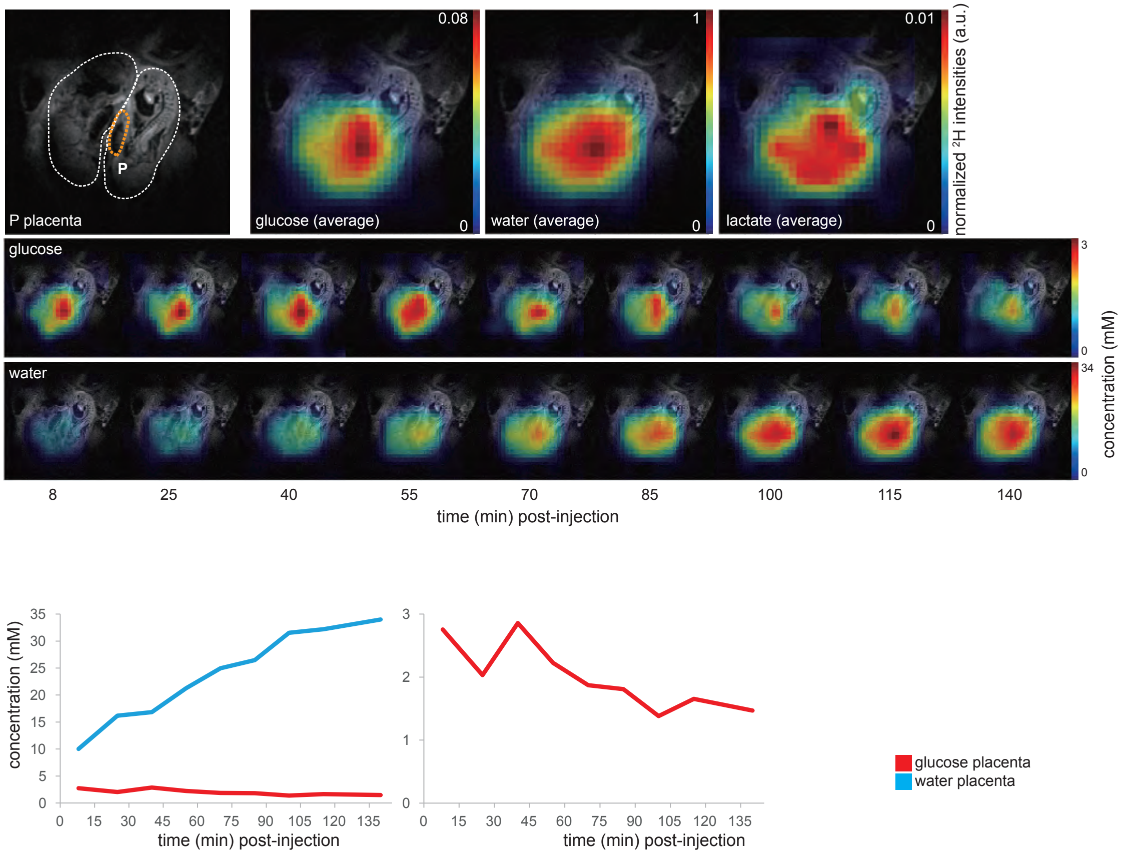

Figure S13: Idem as in Figure S12 but on a different animal. Glucose concentrations are kept below ~3 mM (zoomed on right graph). Water is steadily produced in the placenta rising up to 34 mM. Lactate is produced mainly in the placenta, but at such low levels that it can only be visualized through summation of the time series.

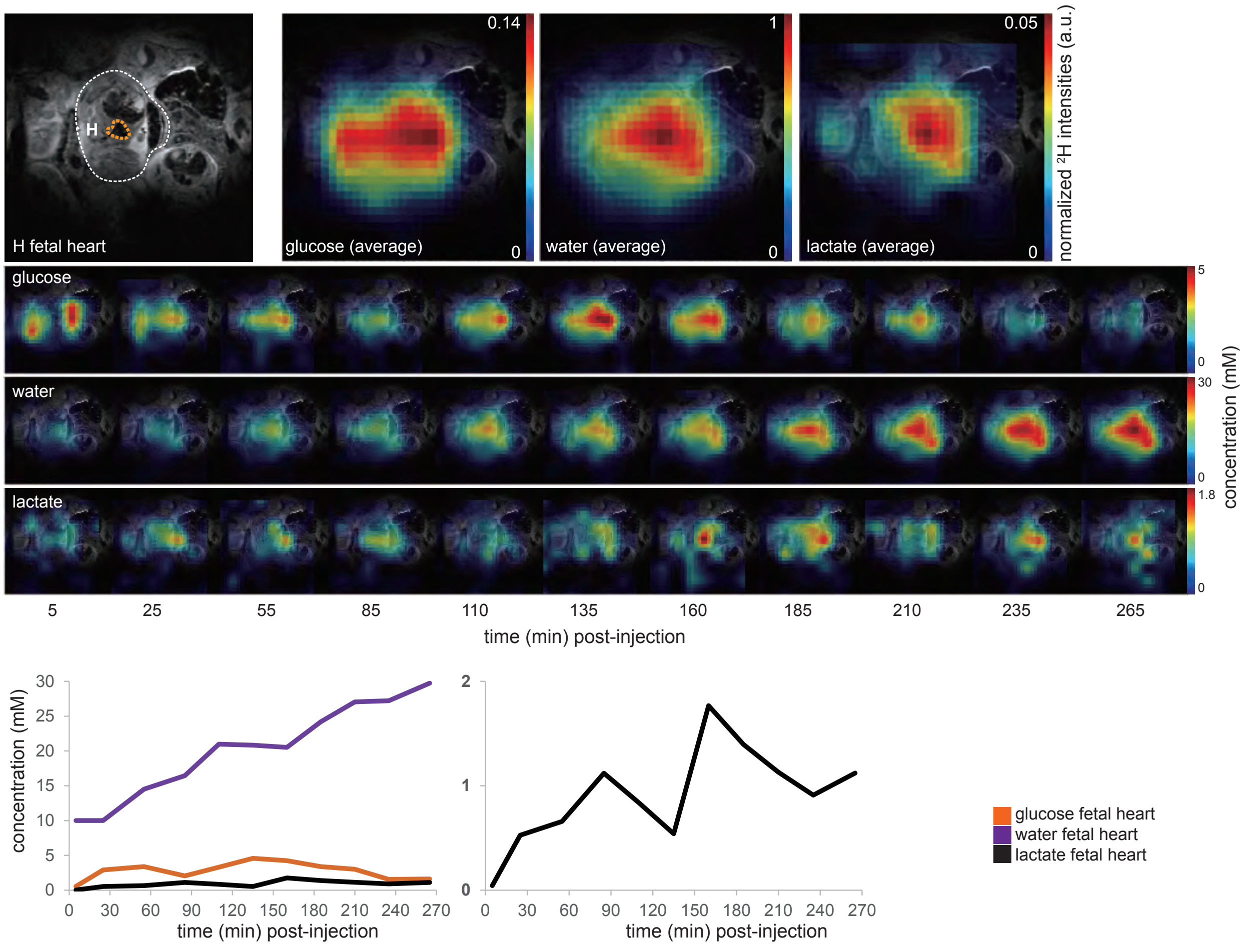

Figure S14: Idem as in Figure S12 but on a different animal. Glucose concentrations are kept ≤5 mM. Water is steadily produced in the fetus (heart?) rising up to 30 mM. Lactate is produced mainly in the fetus (apparently the heart) up to concentrations of 1.8 mM (zoom on right graph).

Supporting Information S4. DMI analyses on l-NAME-treated animals (bolus injections)

This section presents the time-series of ^{2}H MRSI maps collected after an intravenous bolus of $^{2}H_{6,6'}$-glucose was administered to the n=9 pregnant mice that had been treated with l-NAME at a dose of 20 mg/kg_body_weight/day for two days, as described in Methods. All ^{2}H MRSI images were recorded as described in the main text over a time course >2 h. In all cases the top rows present an anatomical reference image indicating organs and the time-averaged metabolic maps resulting from the study for $^{2}H_{6,6'}$-glucose and its metabolic products ^{2}H-water and $^{2}H_{3,3'}$-lactate. Their colormaps are normalized to the maximum signal (water). Shown underneath these spectra are the series of site-resolved ^{2}H MR images collected in the exam overlaid with anatomical ^{1}H MR references, as well as time traces for individual organs of interest as depicted below the images (as defined within the limits of our spatial resolution).

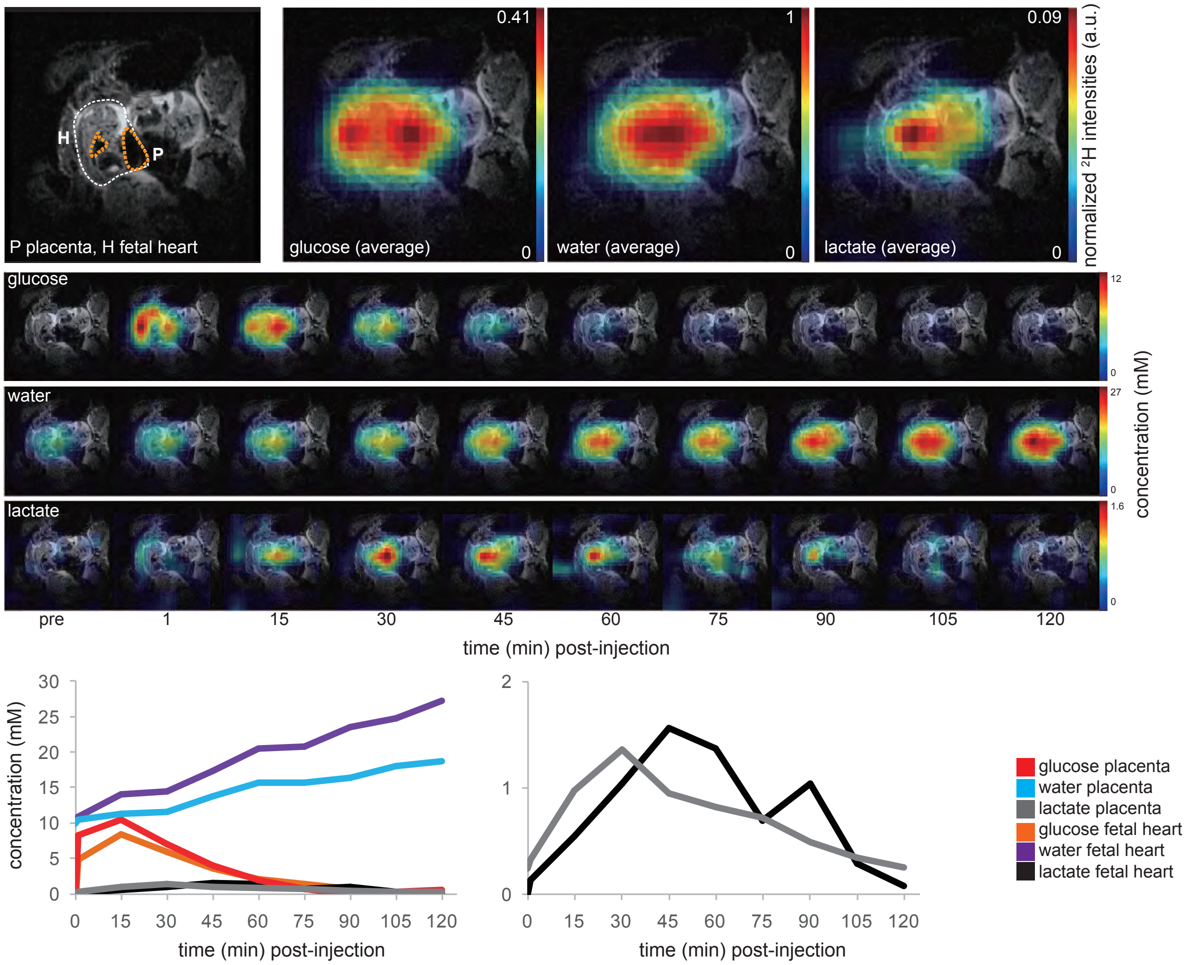

Figure S15: Akin to Figure 3 in the main text. Glucose is initially perfusing in the placenta of this l-NAME-treated pregnant mouse, peaking 15-23 min after injection at 11 mM, and into the fetus (liver?) with a peak at ~20 min after injection of 9 mM. Water is steadily produced in the placenta rising to 17 mM and in the fetal liver rising to 27 mM. Lactate is produced in the placenta with a peak at 30-40 min of 1.4 mM and in the fetus (liver?) with a peak at 45-55 min of 1.6 mM.

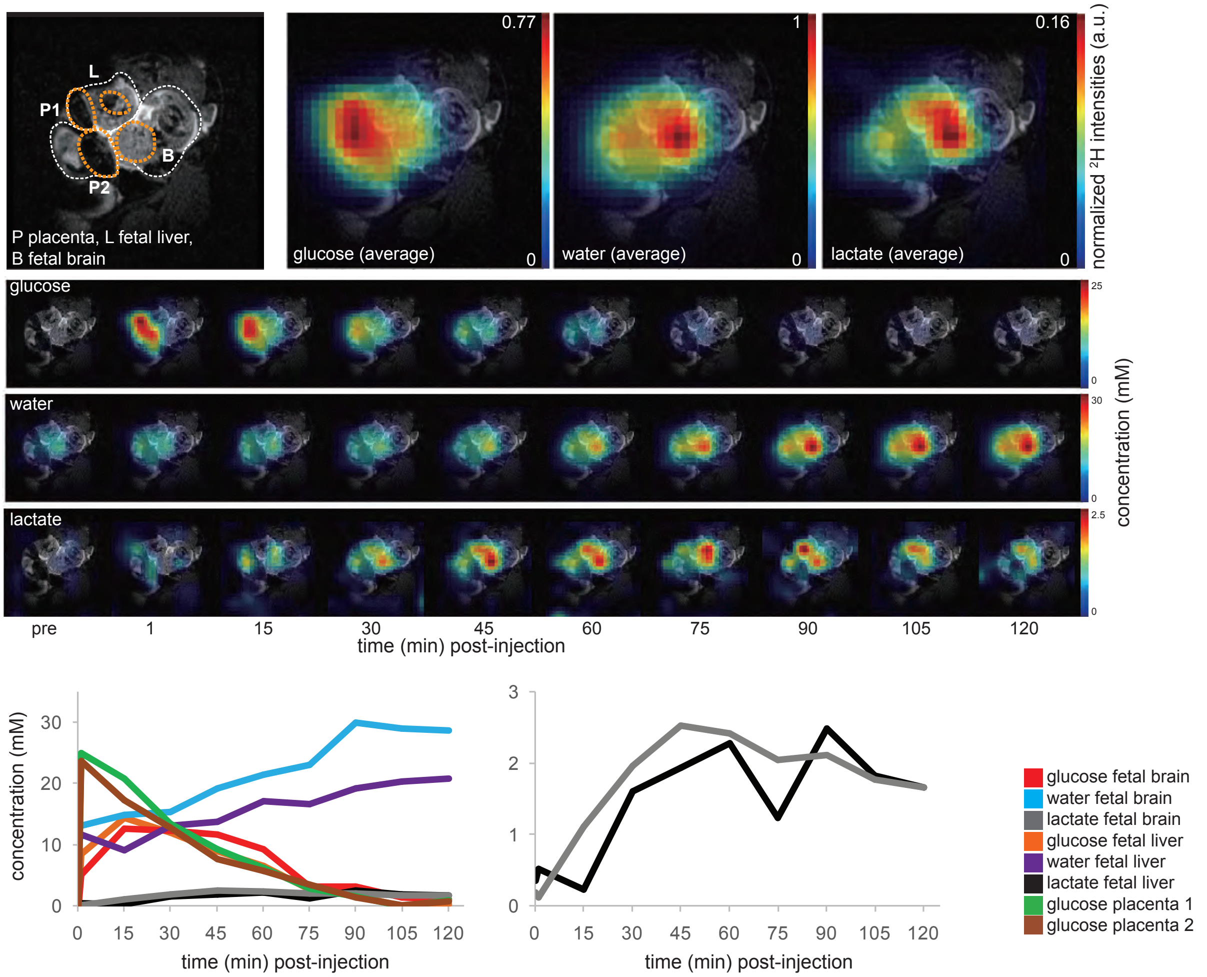

Figure S16: Idem as in Figure S15 but on a different animal. Glucose is initially perfusing two placentas with a peak immediately after injection of 25 mM, in the fetus (liver and brain) with peak at 15-25 min after injection of ~13-15 mM. Water is steadily produced in the fetal liver rising to 21 mM and in the fetal brain rising to 30 mM. Lactate is produced in the fetus (liver?) with a peak at ≥60 min of 2.4 mM. Lactate also appears produced in the fetal brain with a peak at 45-55 min of 2.5 mM. Lactate in fetal compartments washes out slowly, with considerable amounts of lactate being detected at the end of the experiment.

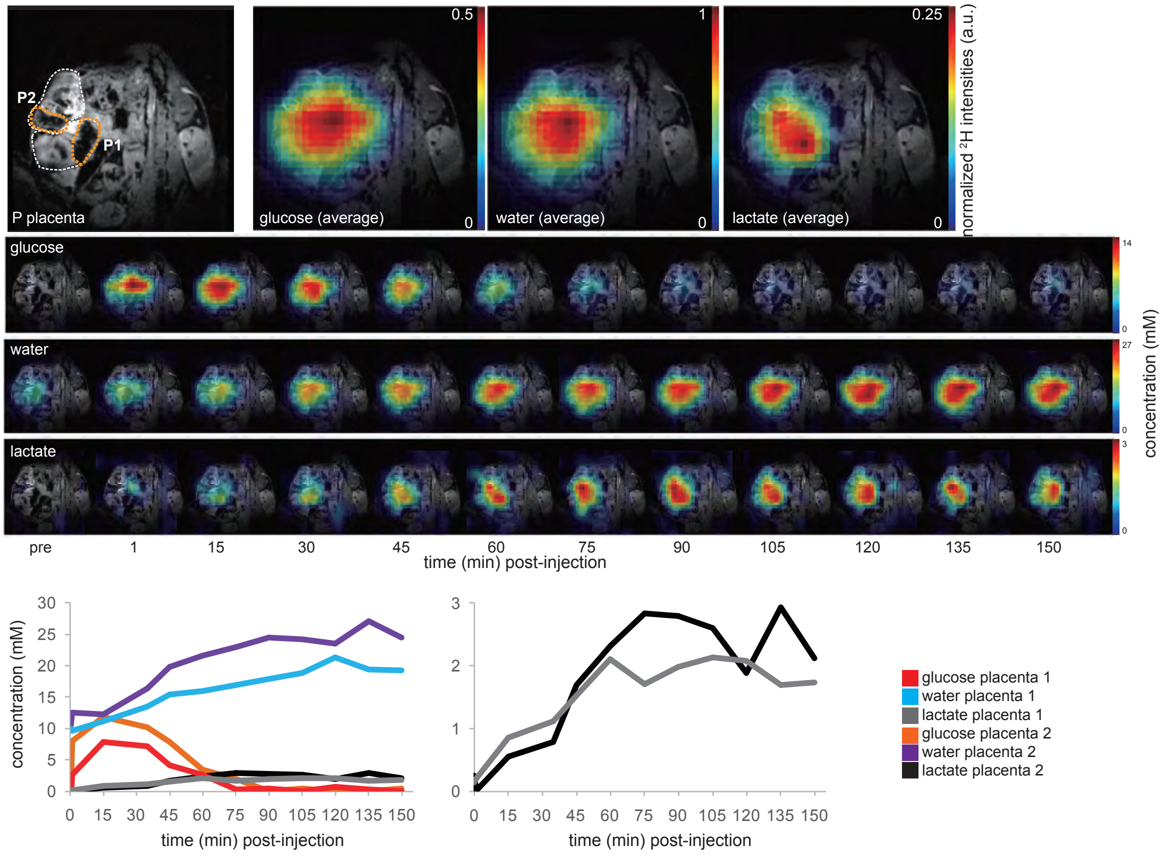

Figure S17: Idem as in Figure S15 but on a different animal. Glucose is initially perfusing the placenta 1 with a peak at ~9 min at 8 mM and placenta 2 with a peak right after injection at 12 mM. Water is steadily produced in the placenta 1 rising to 20 mM and placenta 2 rising to 27 mM. Lactate is produced in placenta 1 with a peak at 60-68 min of 2 mM and in placenta 2 with a peak at 75-85 min of 3 mM. Lactate in both placentas barely washes out, with considerable amounts of lactate being detected at the end of the experiment.

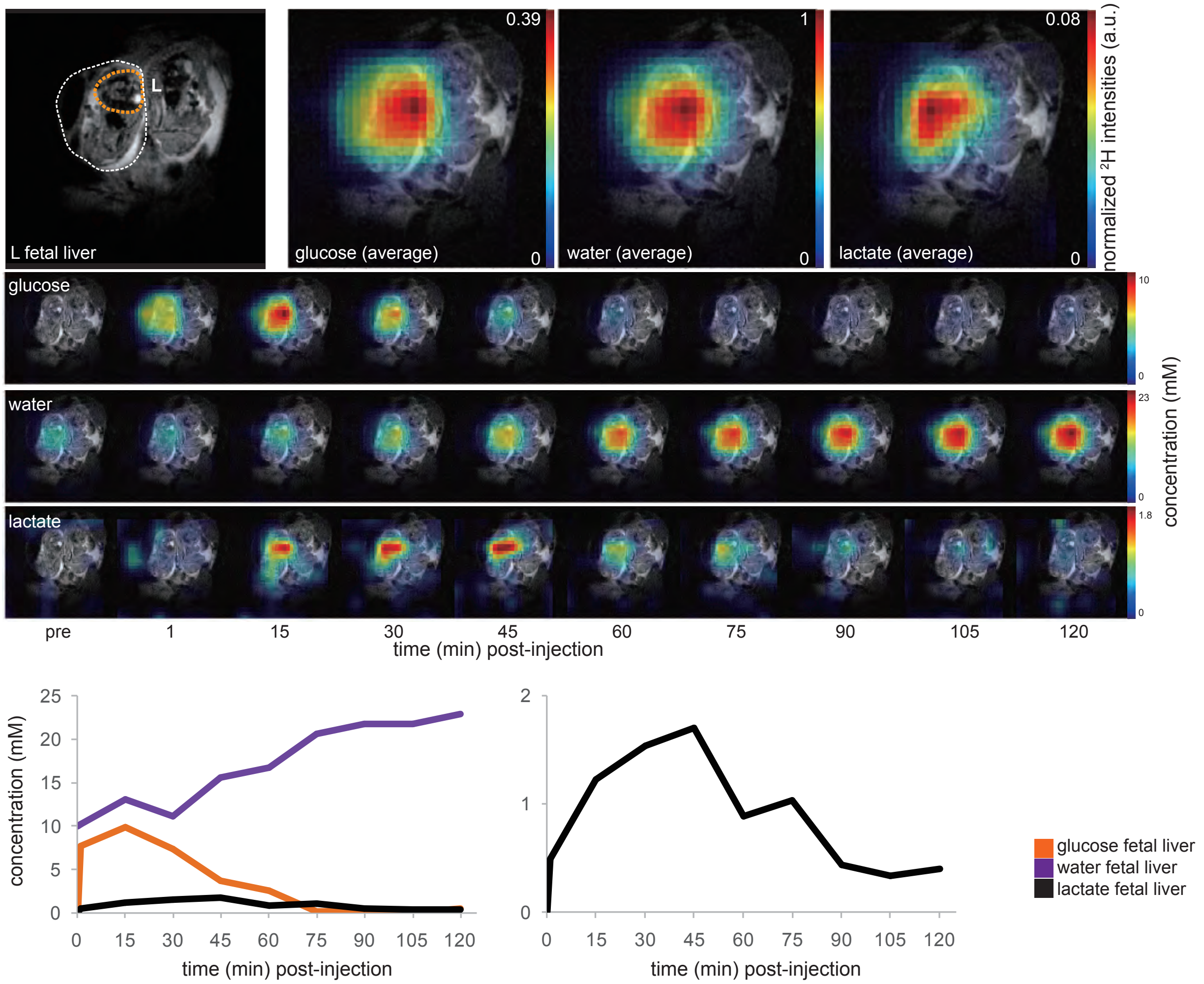

Figure S18: Idem as in Figure S15 but on a different animal. Glucose is initially perfusing the fetus (liver?) with a peak at 15-20 min after injection of 10 mM. Water is steadily produced in the fetal liver rising to 23 mM. Lactate is produced in the fetus with a peak at ~45 min of 1.8 mM.

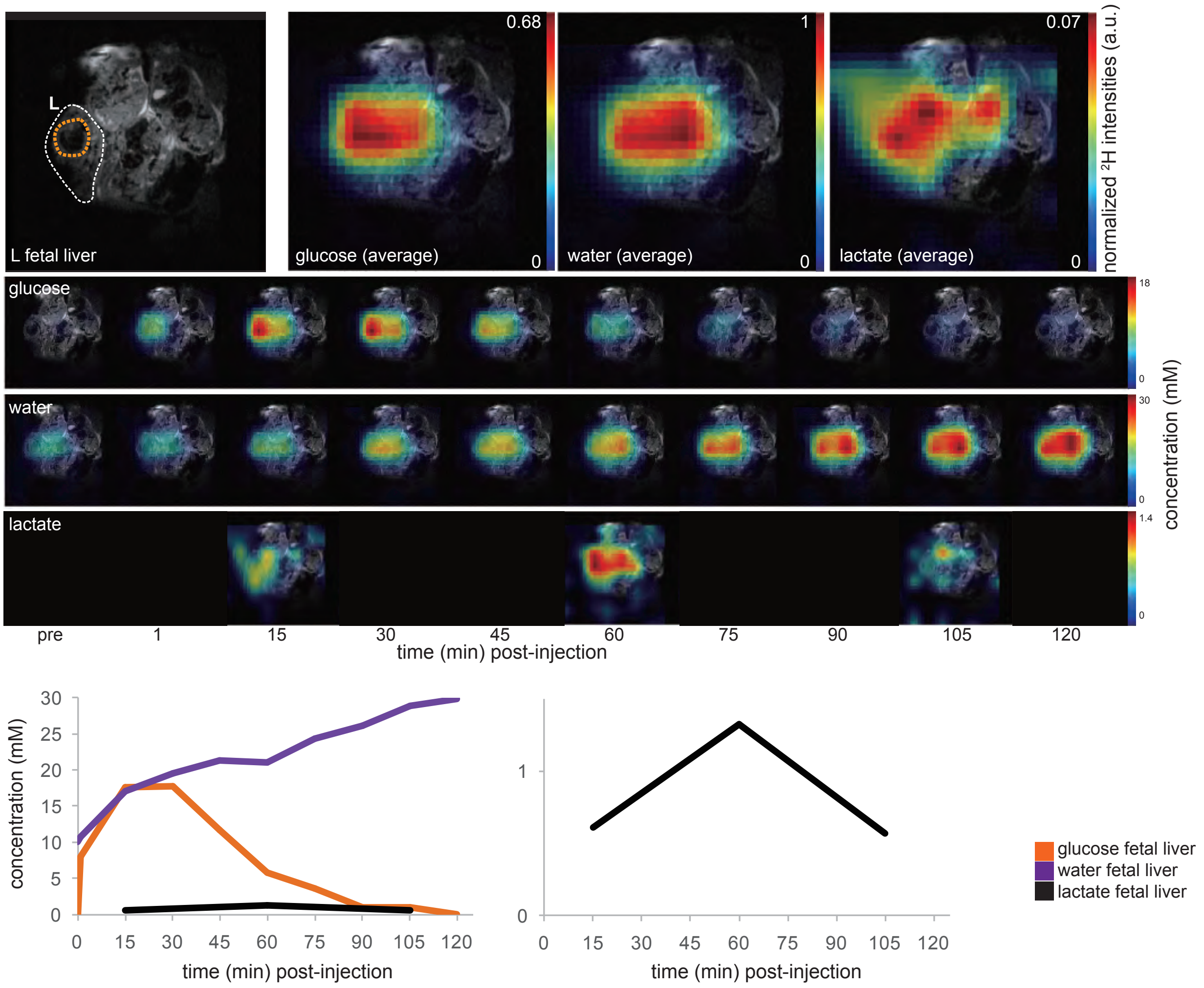

Figure S19: Idem as in Figure S15 but on a different animal. Glucose is initially perfusing the fetus with a peak at 15-25 min after injection of 18 mM. Water is steadily produced in in the fetal liver rising to 30 mM. Lactate is produced in the fetus (liver?) with a peak at ~60 min of 1.4 mM.

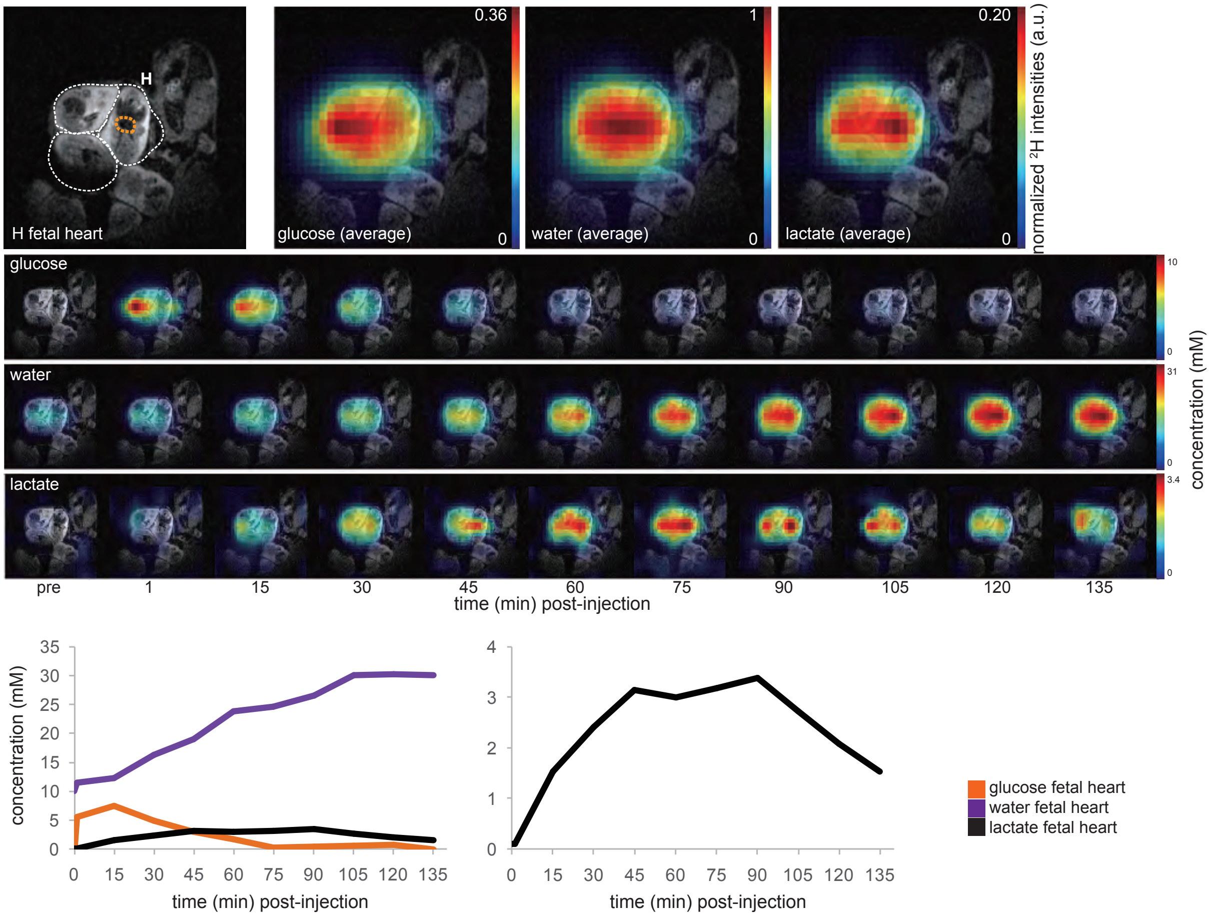

Figure S20: Idem as in Figure S15 but on a different animal. Glucose is initially perfusing the fetus (heart?) with a peak at ~15 min after injection of 7 mM. Water is steadily produced in the fetal heart rising to 31 mM. Lactate is produced in the fetus (heart?) with a peak at 45-90 min of 3.4 mM.

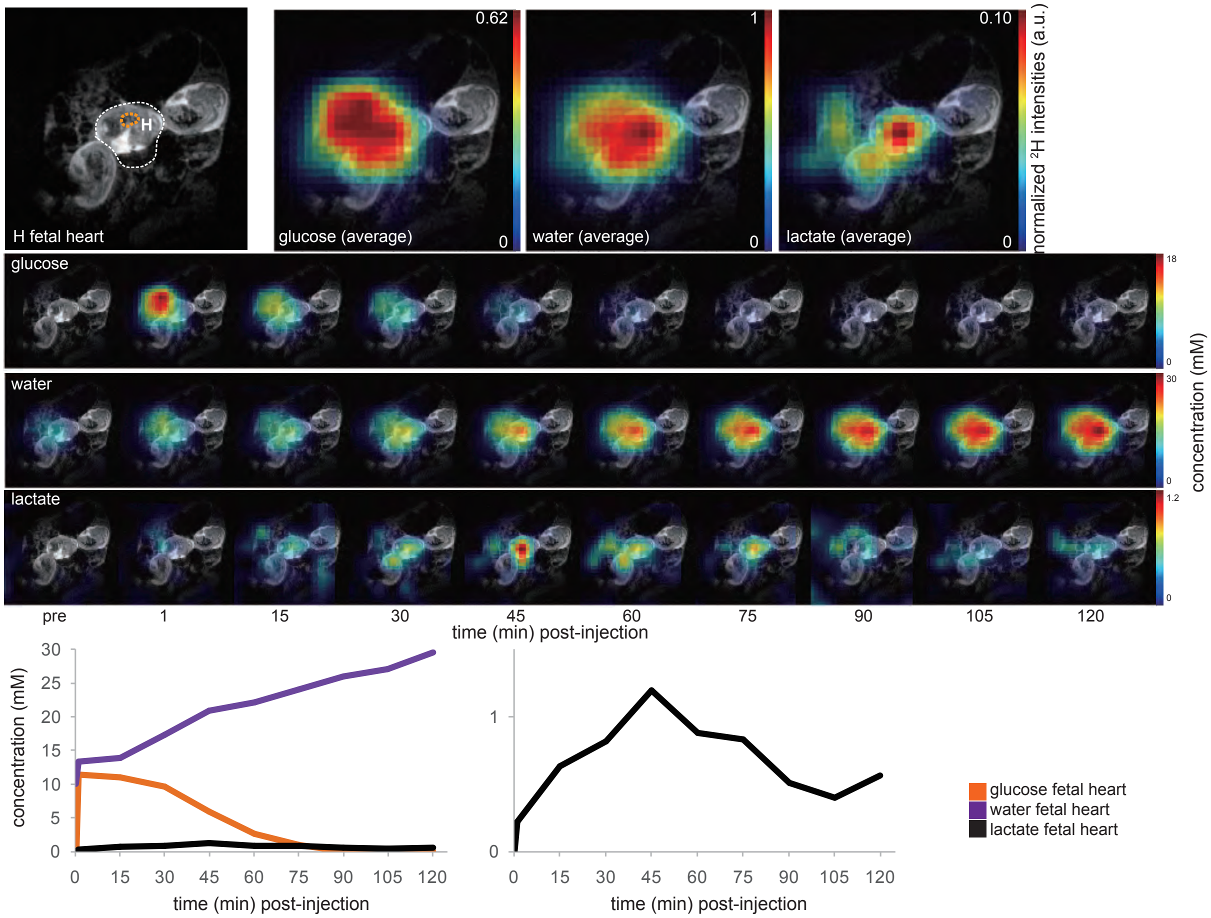

Figure S21: Idem as in Figure S15 but on a different animal. Glucose is initially perfusing the fetus with a peak at right after injection of 12 mM. Water is steadily growing in the fetus (apparently in the heart), rising to 30 mM. Lactate is produced in the fetus peaking at ~45 min with 1.2 mM.

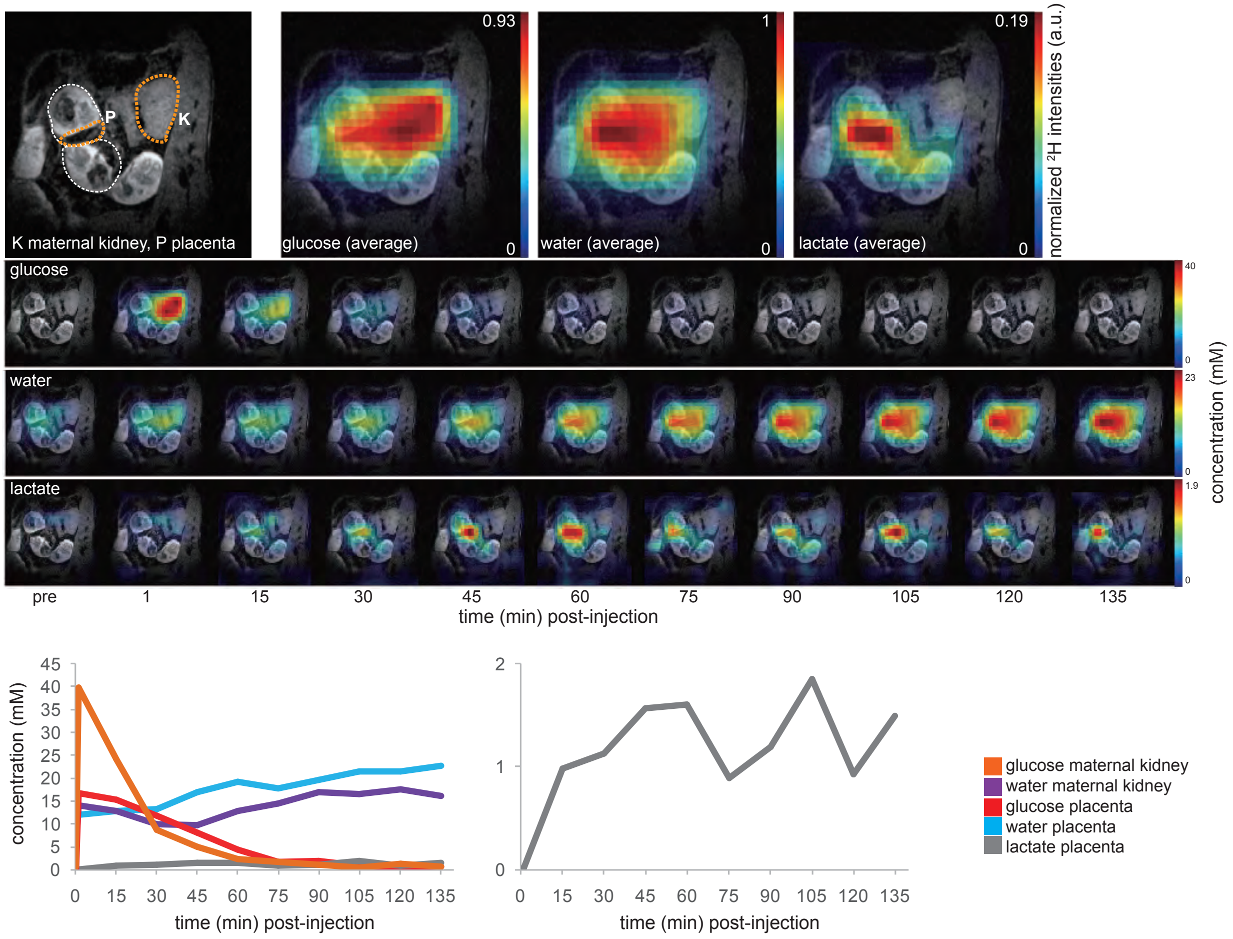

Figure S22: Idem as in Figure S15 but on a different animal. Glucose is initially perfusing the maternal kidney with a peak at 1-9 min after injection at 40 mM and the placenta with a peak at 1-9 min after injection at 17 mM. Water is steadily produced in the placenta rising to 23 mM. Lactate is produced in placenta with a peak at 60-90 min up to 1.9 mM.

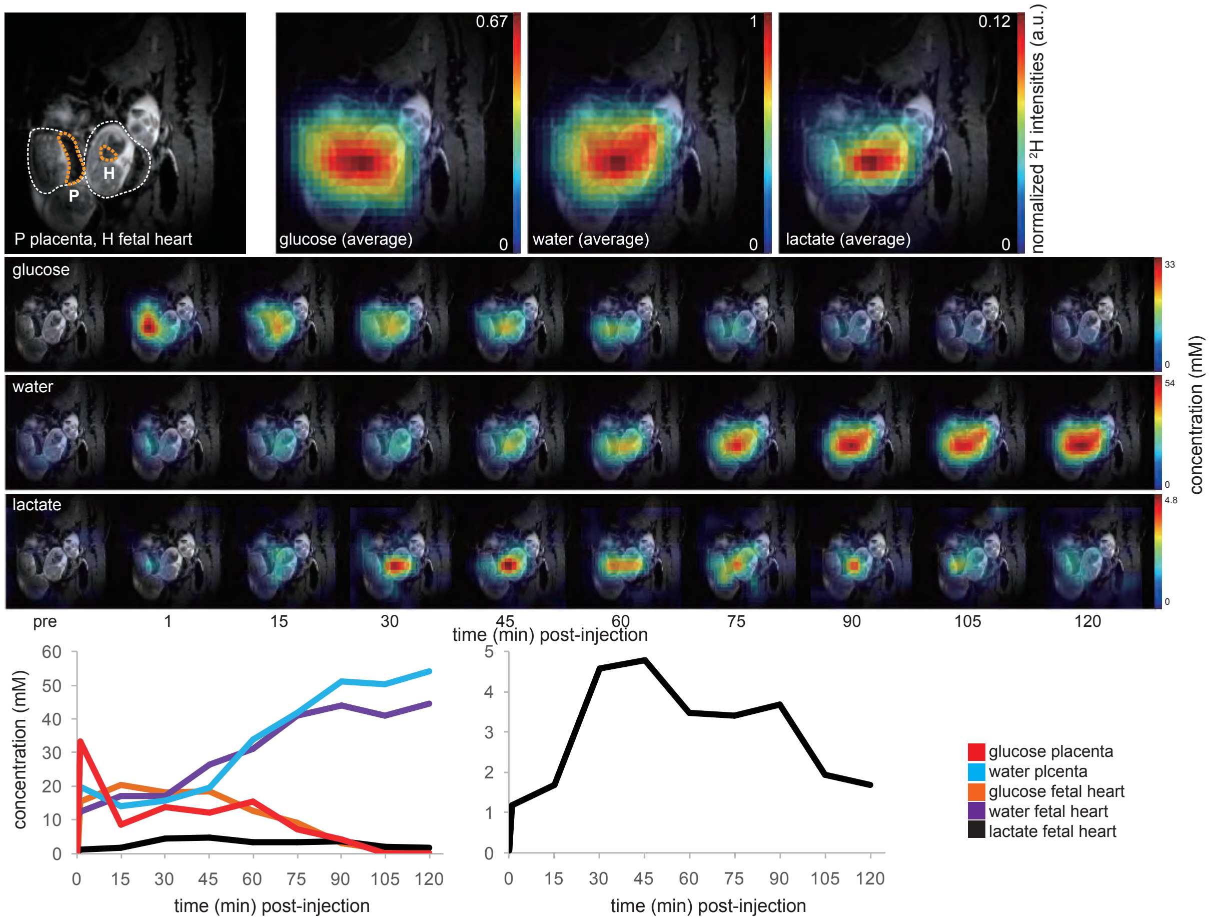

Figure S23: Idem as in Figure S15 but on a different animal. Glucose is initially perfusing the placenta with a peak at ~1 min at 33 mM and the fetus (heart?) at ~20 min after injection at 21 mM. Water is steadily produced in the fetus rising to 43 mM and in the placenta up to 54 mM. Lactate is produced in the fetus (presumably in the heart) with a peak at ~45min of 4.8 mM.

Supporting Information S4. Ancillary ^{2}H analysis

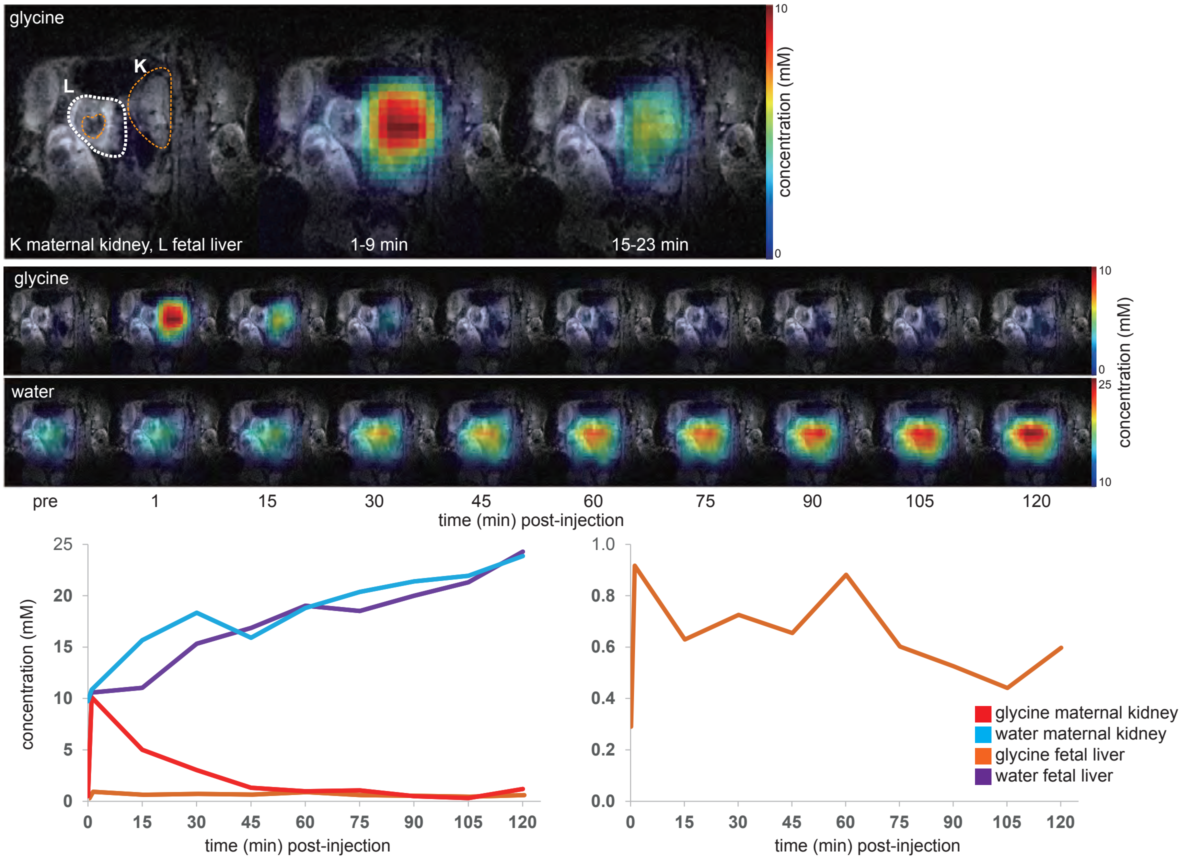

Figure S24: Akin to Figure 2 in the main text, but after intravenous administration of $^2H_{2,2'}$-glycine to a control pregnant mouse. The ^{2}H MRSI images for $^2H_{2,2'}$-glycine and its metabolic product ^{2}H-water were recorded over a time course ≥2 h. The top row depicts an anatomical reference image and the first two time series $^2H_{2,2'}$-glycine images. Time-series of ^{2}H MRSI maps and corresponding time traces for individual organs of interest are depicted below. Gycine is initially perfusing the maternal kidney with a peak at 1-9 min after injection at 10 mM. Apart from in the maternal kidney glycine is only found in small amounts in the fetus(liver?) at ≤0.9 mM. Water is steadily produced in the maternal kidney and the fetus, rising to 25 mM. No other metabolic products are observed.

Supporting Information S5: Analytical solutions for Eqs. (1)-(5)

The manuscript puts forward a kinetic model for reproducing the time traces of the DMI experiments, based on the following differential equations

$$\frac{dG_m}{dt} = -k_{out}^{Gm} \cdot G_m \qquad (1) \qquad \frac{dG_f}{dt} = -k_{out}^{Gf} \cdot G_f + k_{in}^{Gtf} \cdot G_m \qquad (2)$$

$$\frac{dW_m}{dt} = -k_{out}^{Wm} \cdot W_m + k_{met}^{Wm} \cdot G_m \qquad (3) \qquad \frac{dW_f}{dt} = -k_{out}^{Wf} \cdot W_f + k_{in}^{Wm} \cdot W_m + k_{met}^{Wf} \cdot G_f \quad (4)$$

$$\frac{dL_f}{dt} = -k_{out}^{Lf} \cdot L_f + k_{met}^{Lf} \cdot G_f \qquad (5) \quad .$$

where the various variables are described in the main text. Analytical solutions for these simultaneous equations were found for the boundary conditions $W_m(0) = W_f(0) = 10$ mM, $G_f(0) = L_f(0) = 0$. These solutions were used in fitting the experimental data using Maple code (available upon request), and they were

$$G_m(t) = G_o \cdot e^{-k_{out}^{Gm} \cdot t} \qquad (6)$$

$$G_f(t) = \left(\frac{k_{in}^{Gf} \cdot G_o}{k_{out}^{Gm} - k_{out}^{Gf}}\right)\left(e^{-k_{out}^{Gf} \cdot t} - e^{-k_{out}^{Gm} \cdot t}\right) \qquad (7)$$

$$W_m(t) = \left[G_o \cdot k_{met}^{Wm}\left(1 - e^{-k_{out}^{Gm} \cdot t + k_{out}^{Wm} \cdot t}\right) + W(0)(k_{out}^{Gm} - k_{out}^{Wm})\right] \frac{e^{-k_{out}^{Wm} \cdot t}}{k_{out}^{Gm} - k_{out}^{Wm}} \qquad (8)$$

$$L_f(t) = k_{in}^{Gf} \cdot G_o \cdot k_{met}^{Lf}\left(\frac{(k_{out}^{Gm} - k_{out}^{Gf})e^{-k_{out}^{Gm} \cdot t}}{k_{out}^{Lf} - k_{out}^{Gm}} + \frac{\left(k_{out}^{Gf} - k_{out}^{Gm}\right) \cdot e^{-k_{out}^{Gf} \cdot t}}{k_{out}^{Lf} - k_{out}^{Gf}}\right) + \frac{e^{-k_{out}^{Lf} \cdot t}}{\left(k_{out}^{Lf} - k_{out}^{Gf}\right) \cdot \left(k_{out}^{Lf} - k_{out}^{Gm}\right)}) \qquad (9)$$

$$W_f(t) = e^{-k_{out}^{Wm} \cdot t} \text{ x}$$

$$\Bigg[\frac{\left(k_{met}^{Wm} \cdot G_o + W(0) \cdot \left(k_{out}^{Gm} - k_{out}^{Wf}\right)\right) \cdot k_{in}^{Wm} \cdot \left(k_{out}^{Gf} - k_{out}^{Wf}\right) + \left(k_{met}^{Wm} \cdot k_{out}^{Wm} - k_{met}^{Wf} \cdot k_{out}^{Wf}\right) \cdot k_{in}^{Gf} \cdot G_o + W(0) \cdot k_{out}^{Wf} \cdot \left(k_{out}^{Wf} - k_{out}^{Gm} - k_{out}^{Gf}\right) \cdot \left(k_{out}^{Gm} - k_{out}^{Wf}\right)}{\left(k_{out}^{Wm} - k_{out}^{Wf}\right) \cdot \left(k_{out}^{Gf} - k_{out}^{Wf}\right) \cdot \left(k_{out}^{Gm} - k_{out}^{Wf}\right)} +$$

$$\frac{1}{(k_{out}^{Wm} - k_{out}^{Gm})(k_{out}^{Gf} - k_{out}^{Gm})}\left(\frac{G_o \cdot e^{-k_{out}^{Gm} \cdot t}}{(k_{out}^{Wf} - k_{out}^{Gm})}\left(k_{in}^{Gf} \cdot k_{met}^{Wf} \cdot k_{out}^{Wm} - k_{in}^{Gf} \cdot k_{met}^{Wf} \cdot k_{out}^{Gm} - k_{in}^{Wm} \cdot k_{met}^{Wm} \cdot k_{out}^{Gm} + k_{in}^{Wm} \cdot k_{met}^{Wm} \cdot \right.\right.$$

$$\left.k_{out}^{Gf}\right) + \frac{k_{in}^{Gf} \cdot k_{met}^{Wf} \cdot G_o \cdot e^{-k_{out}^{Gm} \cdot t}}{(k_{out}^{Wf} - k_{out}^{Gf})}\left(\frac{\left(k_{out}^{Gm}\right)^2}{\left(k_{out}^{Gm} - k_{out}^{Wm}\right)} - \frac{k_{out}^{Gm} \cdot k_{out}^{Gf}}{\left(k_{out}^{Gm} - k_{out}^{Gf}\right)} - \frac{k_{out}^{Wm} \cdot k_{out}^{Gm}}{\left(k_{out}^{Gm} - k_{out}^{Gf}\right)} + \frac{k_{out}^{Wm} \cdot k_{out}^{Gf}}{\left(k_{out}^{Gm} - k_{out}^{Gf}\right)}\right) +$$

$$\frac{k_{in}^{Wm}}{(k_{out}^{Gm} - k_{out}^{Wm})\left(k_{out}^{Wf} - k_{out}^{Wm}\right)}\left(\left(k_{met}^{Wm} \cdot G_o + W(0) \cdot (k_{out}^{Gm} - k_{out}^{Wm})\right)\left((k_{out}^{Gm})^2 \cdot e^{-k_{out}^{Gf} \cdot t} - k_{out}^{Gm} \cdot k_{out}^{Gf} \cdot e^{-k_{out}^{Wm} \cdot t} - \right.\right.$$

$$\left.\left.\left. k_{out}^{Wm} \cdot k_{out}^{Gm} \cdot e^{-k_{out}^{Wm} \cdot t} + k_{out}^{Wm} \cdot k_{out}^{Gf} \cdot e^{-k_{out}^{Wm} \cdot t}\right)\right)\right)\Bigg] \qquad (10)$$

Supporting Information S6. Spectrally-resolved kinetic ^{2}H MR fits for all organs in the control and l-NAME-treated animals analyzed in this study (Figures S3-S12 and S13-S21)

The panels in the following multi-page Figure summarize the kinetic fits for the aforementioned datasets, based on the solutions provided in Supporting Information S5, as specified:

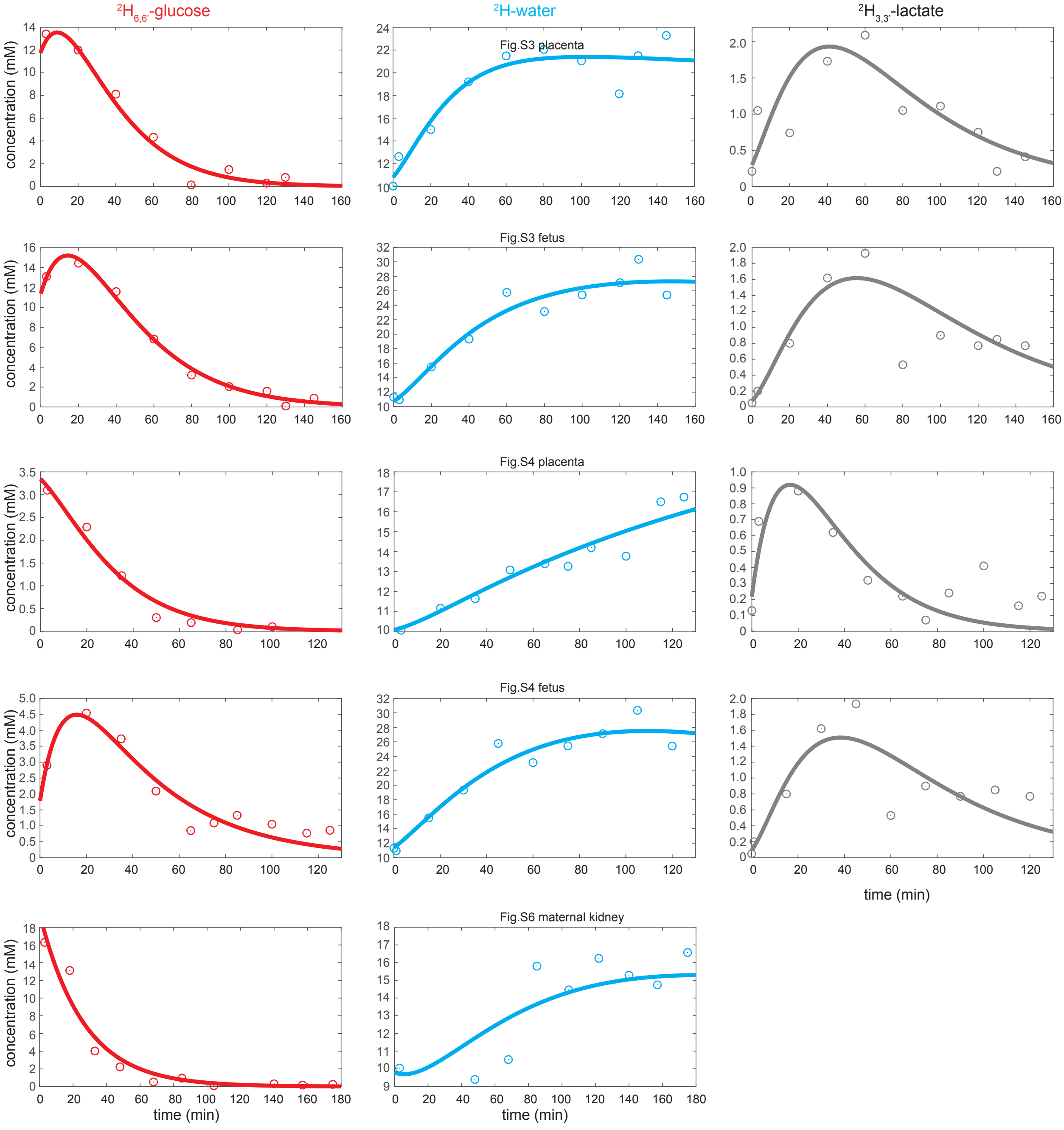

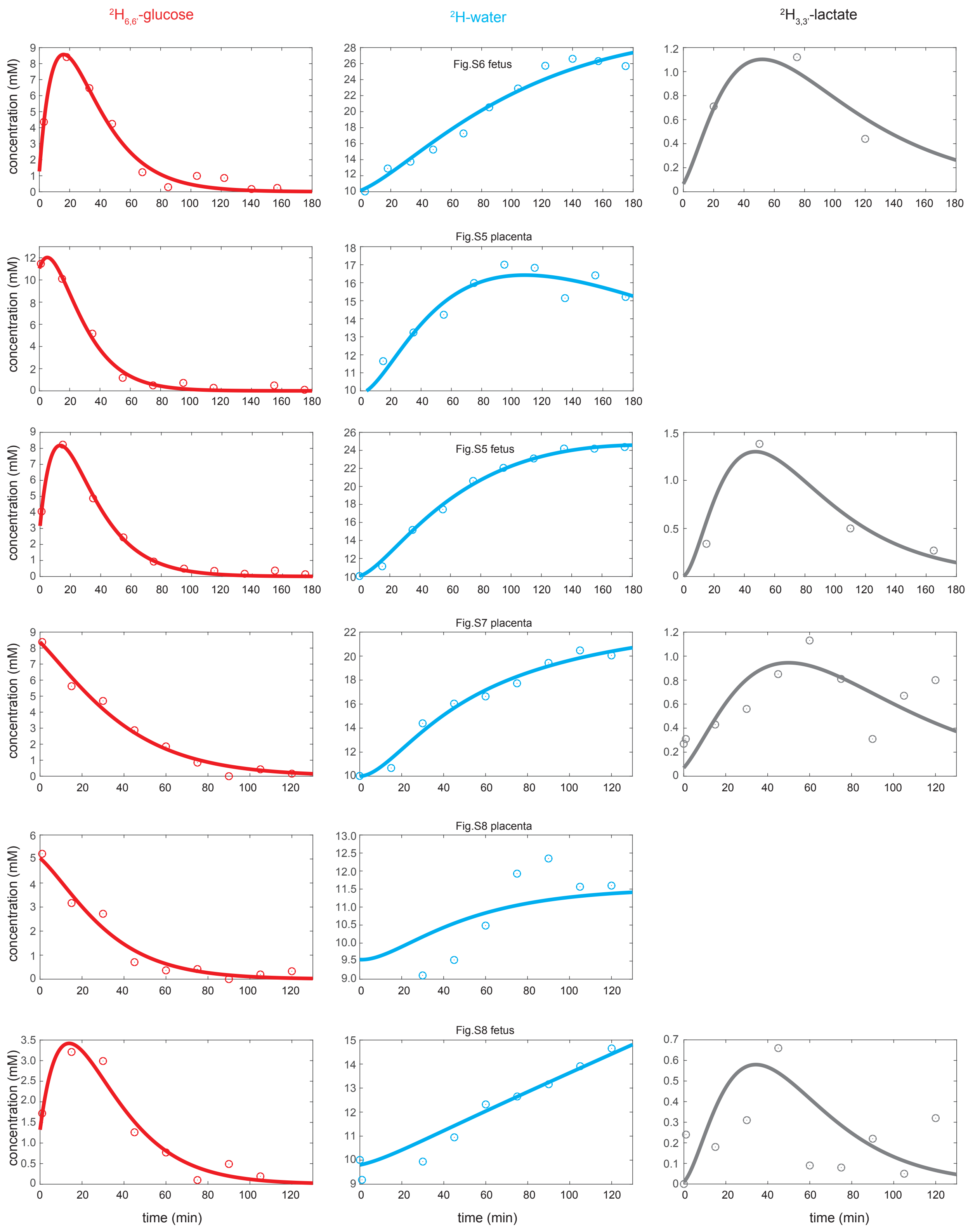

²H6,6'-glucose
²H-water
²H3,3'-lactate
Fig.S6 fetus
Fig.S5 placenta
Fig.S5 fetus
Fig.S7 placenta
Fig.S8 placenta
Fig.S8 fetus
concentration (mM)
time (min)

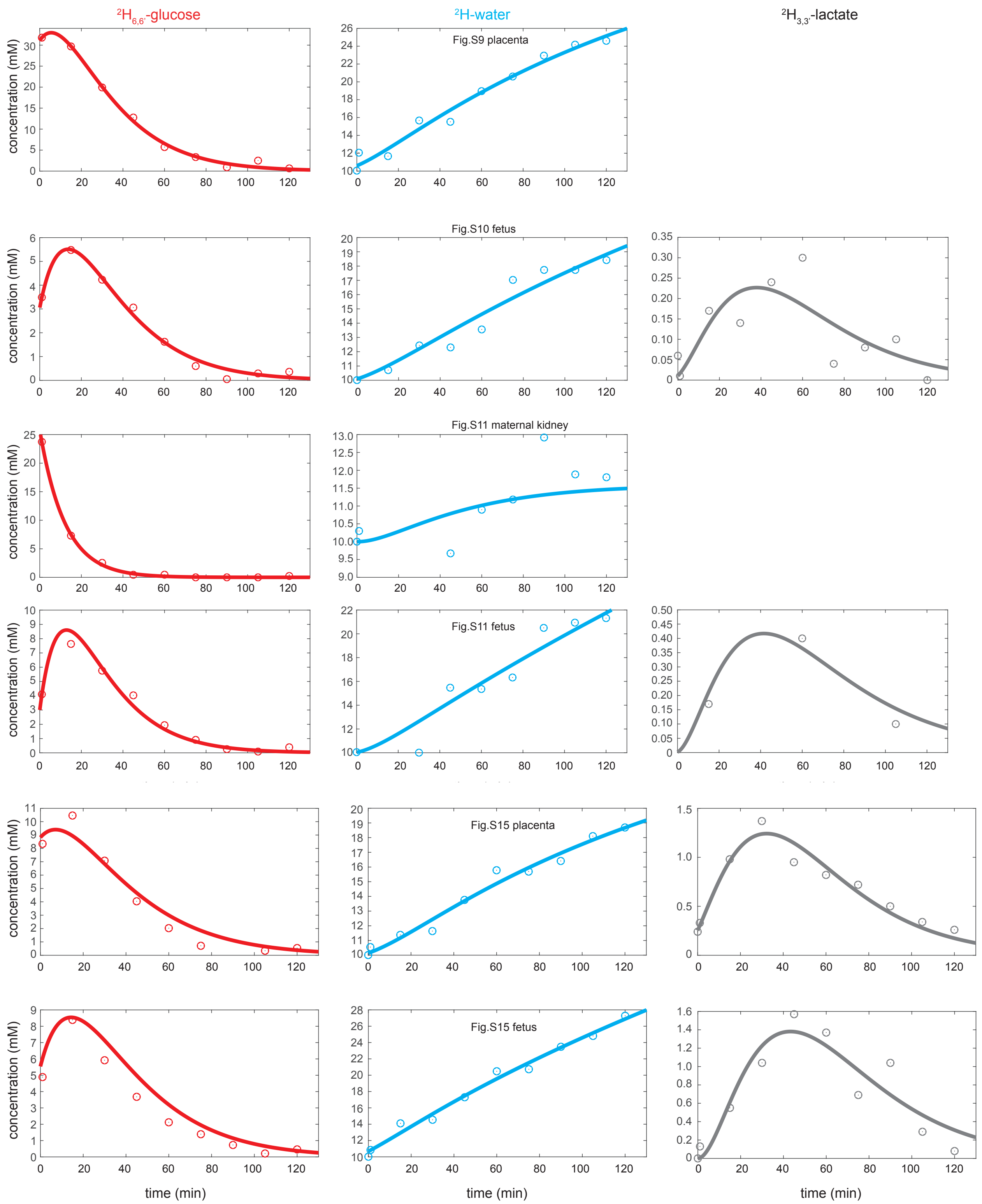

$^2H_{6,6'}$-glucose
^2H-water
$^2H_{3,3'}$-lactate
Fig.S9 placenta
Fig.S10 fetus
Fig.S11 maternal kidney
Fig.S11 fetus
Fig.S15 placenta
Fig.S15 fetus
concentration (mM)
time (min)

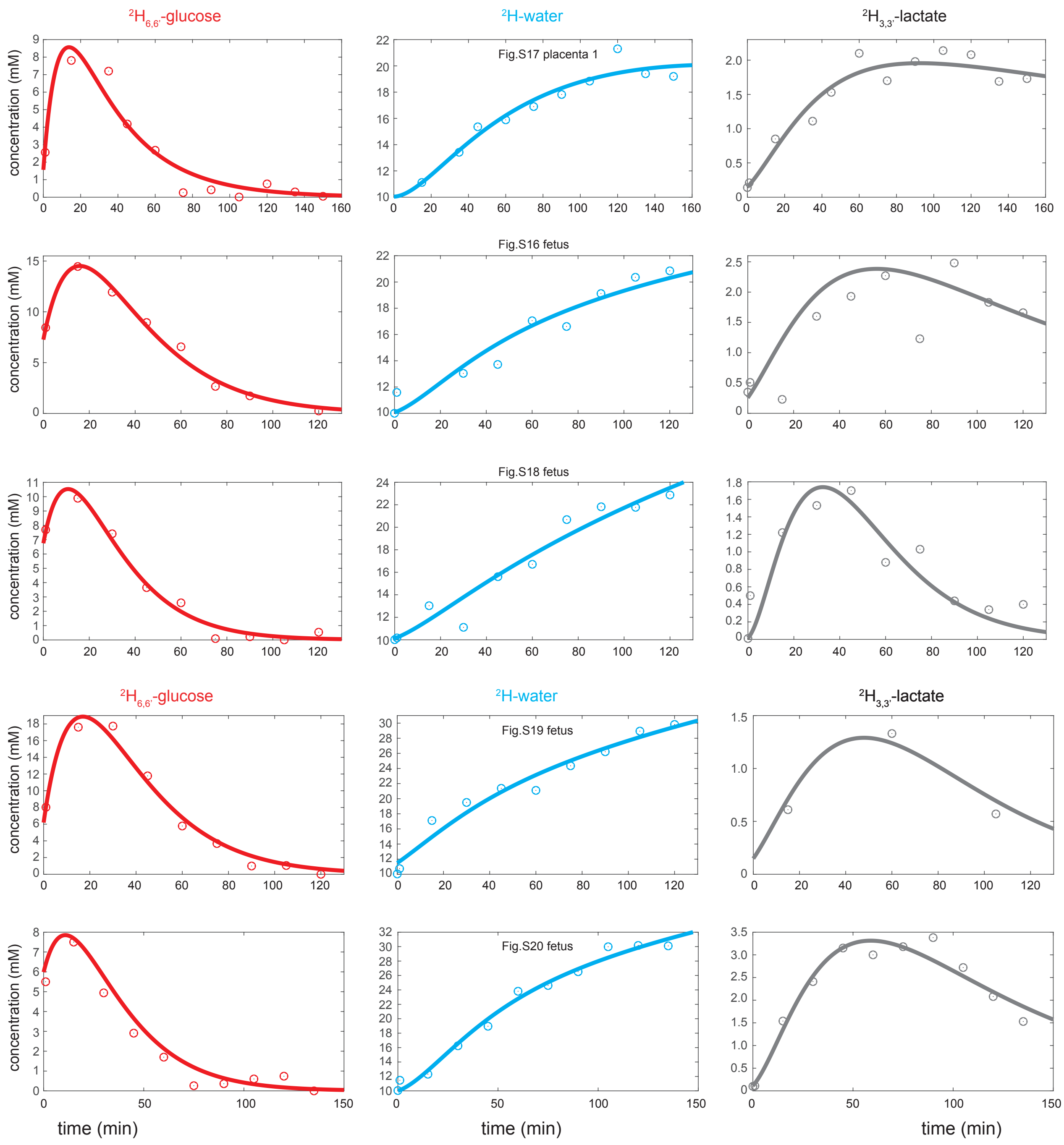

²H6,6'-glucose
²H-water
²H3,3'-lactate
Fig.S17 placenta 1
Fig.S16 fetus
Fig.S18 fetus
²H6,6'-glucose
²H-water
²H3,3'-lactate
Fig.S19 fetus
Fig.S20 fetus
concentration (mM)
time (min)
time (min)
time (min)

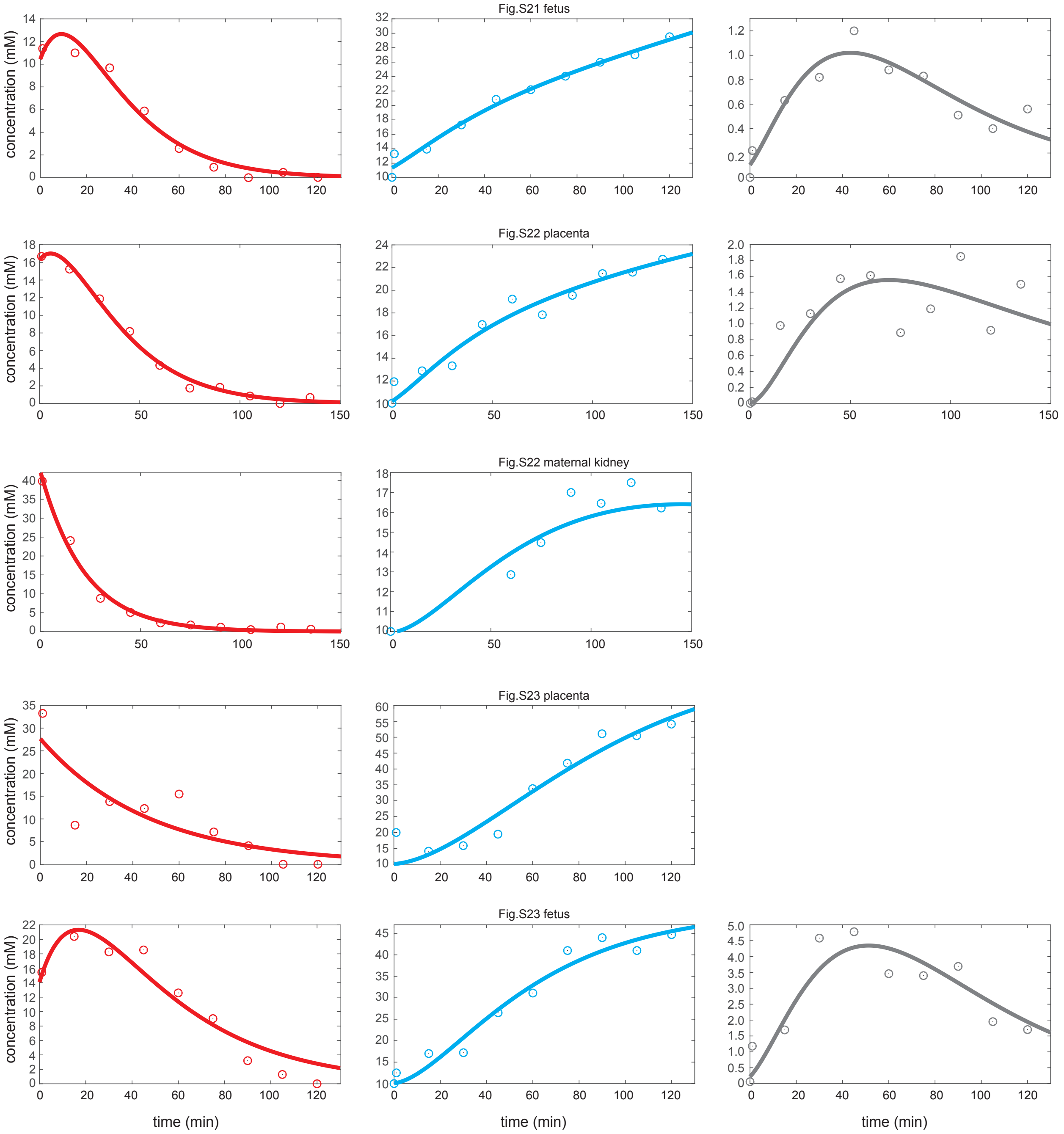

Figure S25: Kinetic fits for datasets in Figures S3-S11 and S15-S23; main fit parameters lead to the data summarized in Table S1.

Supporting Information S7. Kinetic constants arising from the fits presented in Figure S25

Table S1. Average kinetic constants determined for the wildtype control and l-NAME-treated mice derived from the kinetic analyses presented in Supporting Figure S25

	Placenta	***Placenta l-NAME***	***Fetal***	***Fetal l-NAME***	***Maternal kidney***	***Maternal kidney l-NAME***
$G_m(0)$ ***(mM)***	21±6	27±7	17±3	22±5	22±5	42±10
k_{met}^{Wm} ***(mM*min^{-1})***	0.036 ±0.010	0.030 ±0.009	0.032 ±0.006	0.009 ±0.002	0.021 ±0.010	0.005
k_{met}^{Wf} ***(mM*min^{-1})***	0.0063 ±0.0029	0.0029 ±0.0008	0.0039 ±0.0020	0.0078 ±0.0024	-	-
k_{met}^{Lf} ***(mM*min^{-1})***	0.0110 ±0.0032	0.0053 ±0.0014	0.0073 ±0.0025	0.0081 ±0.0019	-	-
k_{out}^{Gm} ***(mM*min^{-1})***	0.052 ±0.004	0.043 ±0.005	0.064 ±0.009	0.047 ±0.005	0.059 ±0.010	0.043 ±0.001
k_{out}^{Gf} ***(mM*min^{-1})***	0.056 ±0.004	0.050 ±0.002	0.052 ±0.007	0.051 ±0.003	-	-
k_{in}^{Gf} ***(mM*min^{-1})***	0.076 ±0.008	0.071 ±0.007	0.065 ±0.003	0.083 ±0.010	-	-
k_{out}^{Lf} ***(mM*min^{-1})***	0.035 ±0.006	0.018 ±0.012	0.030 ±0.006	0.026 ±0.008	-	-
k_{out}^{Wf} ***(mM*min^{-1})***	0.0096 ±0.0029	0.0103 ±0.0027	0.0042 ±0.0016	0.0030 ±0.0008	-	-
k_{out}^{Wm} ***(mM*min^{-1})***	0.0020 ±0.0008	0.0011 ±0.0004	0.0023 ±0.0015	0.0008 ±0.0005	0.0005	~0
k_{in}^{Wm} ***(mM*min^{-1})***	0.011 ±0.002	0.013 ±0.002	0.010 ±0.003	0.012 ±0.002	-	-
